# Risk score for predicting mortality including urine lipoarabinomannan detection in hospital inpatients with HIV-associated tuberculosis in sub-Saharan Africa: Derivation and external validation cohort study

**DOI:** 10.1371/journal.pmed.1002776

**Published:** 2019-04-05

**Authors:** Ankur Gupta-Wright, Elizabeth L. Corbett, Douglas Wilson, Joep J. van Oosterhout, Keertan Dheda, Helena Huerga, Jonny Peter, Maryline Bonnet, Melanie Alufandika-Moyo, Daniel Grint, Stephen D. Lawn, Katherine Fielding

**Affiliations:** 1 TB Centre, London School of Hygiene & Tropical Medicine, London, United Kingdom; 2 Clinical Research Department, London School of Hygiene & Tropical Medicine, London, United Kingdom; 3 Malawi–Liverpool–Wellcome Trust Clinical Research Programme, College of Medicine, University of Malawi, Blantyre, Malawi; 4 Department of Medicine, Edendale Hospital, University of KwaZulu-Natal, Pietermaritzburg, South Africa; 5 Dignitas International, Zomba, Malawi; 6 Department of Medicine, College of Medicine, University of Malawi, Blantyre, Malawi; 7 Centre for Lung Infection and Immunity, University of Cape Town, Cape Town, South Africa; 8 Institute of Infectious Diseases and Molecular Medicine, University of Cape Town, Cape Town, South Africa; 9 Epicentre, Paris, France; 10 Division of Allergology and Clinical Immunology, Department of Medicine, University of Cape Town, Cape Town, South Africa; 11 Institute of Research for Development (IRD), UMI 233 TransVIHMI–UM–INSERM U1175, Montpellier, France; 12 Department of Infectious Disease Epidemiology, London School of Hygiene & Tropical Medicine, London, United Kingdom; 13 University of the Witwatersrand, Johannesburg, South Africa; University of Cape Town, SOUTH AFRICA

## Abstract

**Background:**

The prevalence of and mortality from HIV-associated tuberculosis (HIV/TB) in hospital inpatients in Africa remains unacceptably high. Currently, there is a lack of tools to identify those at high risk of early mortality who may benefit from adjunctive interventions. We therefore aimed to develop and validate a simple clinical risk score to predict mortality in high-burden, low-resource settings.

**Methods and findings:**

A cohort of HIV-positive adults with laboratory-confirmed TB from the STAMP TB screening trial (Malawi and South Africa) was used to derive a clinical risk score using multivariable predictive modelling, considering factors at hospital admission (including urine lipoarabinomannan [LAM] detection) thought to be associated with 2-month mortality. Performance was evaluated internally and then externally validated using independent cohorts from 2 other studies (LAM-RCT and a Médecins Sans Frontières [MSF] cohort) from South Africa, Zambia, Zimbabwe, Tanzania, and Kenya. The derivation cohort included 315 patients enrolled from October 2015 and September 2017. Their median age was 36 years (IQR 30–43), 45.4% were female, median CD4 cell count at admission was 76 cells/μl (IQR 23–206), and 80.2% (210/262) of those who knew they were HIV-positive at hospital admission were taking antiretroviral therapy (ART). Two-month mortality was 30% (94/315), and mortality was associated with the following factors included in the score: age 55 years or older, male sex, being ART experienced, having severe anaemia (haemoglobin < 80 g/l), being unable to walk unaided, and having a positive urinary Determine TB LAM Ag test (Alere). The score identified patients with a 46.4% (95% CI 37.8%–55.2%) mortality risk in the high-risk group compared to 12.5% (95% CI 5.7%–25.4%) in the low-risk group (*p <* 0.001). The odds ratio (OR) for mortality was 6.1 (95% CI 2.4–15.2) in high-risk patients compared to low-risk patients (*p <* 0.001). Discrimination (c-statistic 0.70, 95% CI 0.63–0.76) and calibration (Hosmer-Lemeshow statistic, *p =* 0.78) were good in the derivation cohort, and similar in the external validation cohort (complete cases *n =* 372, c-statistic 0.68 [95% CI 0.61–0.74]). The validation cohort included 644 patients between January 2013 and August 2015. Median age was 36 years, 48.9% were female, and median CD4 count at admission was 61 (IQR 21–145). OR for mortality was 5.3 (95% CI 2.2–9.5) for high compared to low-risk patients (complete cases *n =* 372, *p <* 0.001). The score also predicted patients at higher risk of death both pre- and post-discharge. A simplified score (any 3 or more of the predictors) performed equally well. The main limitations of the scores were their imperfect accuracy, the need for access to urine LAM testing, modest study size, and not measuring all potential predictors of mortality (e.g., tuberculosis drug resistance).

**Conclusions:**

This risk score is capable of identifying patients who could benefit from enhanced clinical care, follow-up, and/or adjunctive interventions, although further prospective validation studies are necessary. Given the scale of HIV/TB morbidity and mortality in African hospitals, better prognostic tools along with interventions could contribute towards global targets to reduce tuberculosis mortality.

## Author summary

**Why was this study done?:**

- HIV-associated tuberculosis (TB) is very common in hospitals in sub-Saharan Africa, and is a major cause of morbidity and mortality.
- There is a lack of tools to identify which patients are more likely to die early; therefore, these patients cannot be targeted for more intensive clinical care or other treatments in addition to TB antibiotics and antiretroviral drugs.
- A new urine TB diagnostic test (detecting a substance called lipoarabinomannan [LAM]) can identify patients at higher risk of dying and, along with other simply measured clinical signs or symptoms, may be useful for predicting which patients are most likely to do poorly.

**What did the researchers do and find?:**

- We used data from HIV-positive adults admitted to hospital in Malawi and South Africa and diagnosed with TB to develop a simple clinical risk score to identify patients with a 50% chance of dying within 2 months.
- The score included 6 factors that can be measured at admission to hospital, including the results of the urine TB test, presence of anaemia, and some demographic factors.
- We then tested the performance of the risk score using data from studies of different patients from sub-Saharan Africa, and it performed similarly.
- The score was able to identify patients at higher risk of dying during admission to hospital, and after discharge from hospital.

**What do these findings mean?:**

- This score could be used to identify patients admitted to hospital and diagnosed with HIV-associated TB who may benefit most from more intensive clinical care, additional treatments, and/or closer follow-up after discharge.
- It could also be used as a research tool to study new drugs or strategies aimed at reducing mortality from with HIV-associated TB.
- This is the first such tool to our knowledge in this patient population, and future studies could optimise such predictive tools, in particular if appropriate new interventions and/or diagnostics become available.

## Introduction

Tuberculosis (TB) is the leading infectious disease killer globally, causing an estimated 1.7 million deaths globally in 2017 [1]. This burden lies disproportionately in people living with HIV, who account for approximately 1 in 4 TB deaths. The case fatality rate of HIV-associated TB (HIV/TB) is particularly high in hospitals, estimated at 29% in a recent meta-analysis [2]. This may be an underestimate, given that post-mortem studies from sub-Saharan Africa have demonstrated that a high proportion of HIV-positive deaths in facilities have evidence of undiagnosed TB [3].

Interventional studies aiming to reduce mortality in this patient population have demonstrated mortality reductions with improved TB diagnostics [4,5] and appropriately timed initiation of antiretroviral therapy (ART) [6,7]. However, mortality remains substantial despite these interventions, and adjunctive interventions are likely to be needed to further impact mortality. Currently, predictors for mortality are poorly defined. Being able to identify patients at the highest risk of mortality could inform the development and assessment of new interventions, and also identify which patients would benefit most from interventions beyond TB therapy and appropriately timed ART [8].

Clinical decision tools and risk scores are used widely in clinical practice to simplify the identification of patients at highest risk for poor health outcomes. Predictor scores for mortality have been developed for HIV-associated cryptococcal meningitis and pneumonia, and are used to guide management in *Pneumocystis jiroveci* pneumonia [9–11]. Although scores have been developed to predict risk of TB disease in various populations, including TB bacteraemia in hospitalised patients [12], to our knowledge no externally validated scores exist to predict outcomes of TB disease among hospitalised patients with HIV [13,14]. Scores developed to predict TB mortality in settings with low HIV prevalence are also of limited use in people living with HIV due to differences in clinical presentation, pathogenesis, and outcomes [15–18]. A recent study from the US developed and internally validated a score to predict mortality in HIV/TB in low-prevalence settings (US), but this would not be applicable to hospitalised patients in Africa given that many of the variables are not routinely available [19].

We have previously shown that detection of lipoarabinomannan (LAM) in the urine of HIV/TB patients using a cheap (approximately US$3) and quick (testing takes 25 minutes) lateral flow assay is independently associated with a 2- to 3-fold increased risk of mortality [20]. We therefore aimed to investigate if urinary LAM detection, along with other clinical variables readily available in high-burden settings, could be used to predict which HIV-positive patients admitted to hospital and diagnosed with TB were at high risk of early mortality, and to externally validate the predictive tool.

## Methods

### Study design and participants for prediction tool development

We used data from the STAMP (‘rapid urine-based screening for tuberculosis in HIV-positive patients admitted to hospital in Africa’) trial for the clinical risk score derivation [5,21]. The STAMP trial recruited HIV-positive adults (aged 18 years or more), irrespective of symptoms or clinical presentation, who were admitted to medical wards of 2 hospitals in Malawi and South Africa between 26 October 2015 and 19 September 2017. On admission, patients were screened for TB using Xpert MTB/RIF (Xpert; Cepheid) on sputum in both study arms, and Xpert and Determine TB LAM Ag (TB-LAM; Alere) assays on urine in the intervention arm.

Exclusion criteria in the trial were already taking TB treatment and inability to give consent. The clinical teams managing the patients were masked to which TB tests were positive; therefore, management of TB patients should not have differed between arms. The management of HIV/TB in the study hospitals was representative of their local settings and followed local and national guidelines, with no input from the study team (beyond TB diagnostic tests).

Patients diagnosed with TB in the standard-of-care arm had stored urine tested with Xpert and TB-LAM retrospectively. Data were collected at baseline (at or close to admission) on demographics and clinical characteristics, and subsequently on TB investigations and treatment, and clinical events, including death or discharge from hospital. Patients discharged alive were followed up at 2 months by outpatient attendance, home visit, or telephone for vital status. The derivation cohort included all patients (from both trial arms) with laboratory-confirmed TB. The outcome was mortality risk at 2 months after admission. Patients lost to follow-up were assumed alive at 56 days.

### Definitions

Laboratory-confirmed TB was defined as any 1 of a positive smear microscopy, mycobacterial culture, Xpert from any site, or urinary TB-LAM. TB-LAM assay was positive if recorded as ‘grade 1’ or higher on the manufacturer’s (post-2014) reference card. Ability to walk unaided was assessed by healthcare workers (not self-reported by patients), and was equivalent to a Karnofsky functional score below 40 points [22]. WHO danger signs were heart rate > 120 beats per minute, respiratory rate > 30 per minute, temperature > 39°C, and being unable to walk unaided. ‘ART experienced’ was defined as receiving ART at the time of enrolment to the study.

### Score derivation

Candidate predictor variables were identified for inclusion in the predictive model based on a priori clinical knowledge, previous literature, and the need for variables to be objective, reproducible, and available in resource-constrained settings [23]. We considered variables known to be associated with mortality in HIV/TB, including age, sex, ART experience, physiological measurements at admission, weight and/or body mass index, CD4 cell count, functional status (being unable to walk unaided), and haemoglobin [24–28]. Time on ART was not considered as not all patients take ART, and because of challenges in accurately ascertaining duration. Where 2 or more predictors were highly correlated, only 1 was selected, to simplify the prognostic model, as inclusion of all would contribute little additional predictive information [23]. Analyses were planned prospectively (see S1 Appendix) except where indicated as post hoc.

Continuous variables were assessed for non-linearity using fractional polynomials, and categorised based on previously established cutoffs (e.g., CD4 cell count and haemoglobin) or associations with mortality (e.g., age and weight, using the *fp plot* command in Stata). Complete case analysis was chosen for the derivation score as few data (<5%) were missing. We first performed univariable analyses assessing the association of each variable with mortality risk using logistic regression. We then used a backward elimination, stepwise approach to create a multivariable predictive model, starting with all candidate variables, and excluding variables sequentially if *p* > 0.1 using likelihood ratio tests and the Akaike information criterion. Given that there were 94 deaths, we did not want to estimate more than 9 candidate predictors (various studies have shown each candidate predictor studied requires a minimum of 10 events) [29]. Interactions were also assessed using likelihood ratio testing. All analyses were done using Stata version 14, and all *p-*values were 2-sided.

Regression coefficients from the final multivariable model were multiplied by the smallest possible constant and then rounded to the nearest integer, and then assigned as ‘points’ to each variable. The clinical risk score was derived by combining the points based on each patient’s characteristics. High-, medium-, and low-risk groups for mortality were then arbitrarily defined after plotting risk score against observed mortality such that the high-risk group accounted for most (>50%) deaths and the low-risk group accounted for as few deaths as possible.

### Risk score evaluation and internal performance

Mortality risk at 2 months and 95% confidence intervals (CIs) were calculated for each risk group, as were odds ratios (ORs) and 95% CI for mortality. In exploratory analyses, inpatient and outpatient (post-discharge) deaths were also compared between risk groups by restricting analyses to deaths occurring during hospital admission or to deaths occurring after discharge in the subset of patients who were discharged alive from hospital. CD4 cell count and TB-LAM grade were also compared between risk groups. Mortality risk was compared between groups using chi-squared tests.

We assessed the model discrimination (ability to differentiate patients who would die within 2 months and those who would survive) by calculating the concordance index (c-statistic) (also known as the area under the receiver operator curve), assuming a c-statistic < 0.6 showed poor discrimination [30]. Model calibration was assessed by plotting the probability of mortality predicted by the model against observed mortality in the derivation dataset using a calibration plot and the Hosmer-Lemeshow test, assuming a *p <* 0.05 indicated poor calibration. In post hoc analysis, in response to reviewer request and to better understand the utility of the score, the sensitivity and positive predictive value of the score were calculated.

### External validation

To externally validate the clinical risk score, we used data collected independently from 2 studies: (1) a multicentre diagnostic clinical trial of adjunctive urine TB-LAM testing in HIV-positive patients with TB symptoms who were admitted to hospitals in 4 sub-Saharan African countries (South Africa, Zambia, Zimbabwe, and Tanzania) (LAM-RCT) [31] and (2) a prospective cohort study assessing the diagnostic yield of TB-LAM in HIV-positive patients with TB symptoms in Kenya (Médecins Sans Frontières [MSF] cohort study) [32]. Patients were included in the validation cohort if they were adults and had laboratory-confirmed TB (as previously defined). Patients from the LAM-RCT in the ‘no TB-LAM’ arm were excluded, as were outpatients (i.e., patients not admitted to hospital) from the MSF cohort study.

The validation cohort sites were all in settings in sub-Saharan Africa with high HIV prevalence and TB incidence, but differed from the derivation cohort in that all patients had at least 1 TB symptom (cough, fever, weight loss, or night sweats). The LAM-RCT recruitment occurred between 1 January 2013 and 2 October 2014, and the MSF cohort recruitment between 22 October 2013 and 20 August 2015. Mortality outcomes were assessed at 2 months in both studies.

The clinical risk score for mortality was calculated by assigning the same ‘points’ to variables as for the derivation cohort, and the same cutoffs were used to define high-, medium-, and low-risk groups for mortality. Patients with missing observations were excluded, for a complete case analysis. However, sensitivity analyses were done for score performance using multivariate multiple imputation with chained equations for missing data as 42% of patients had missing data in the validation cohort. Data were assumed to be missing at random, and were imputed for missing candidate predictor variables using mortality risk, other candidate predictor variables, and other baseline demographic variables, with 100 imputations.

Evaluation of the score in the validation dataset was done using the same statistical methods as the internal evaluation, with calculation of mortality risk at 2 months, ORs for mortality, and survival curves. Discrimination was assessed using the c-statistic, and calibration with a calibration plot and the Hosmer-Lemeshow test.

The study is reported in concordance with TRIPOD guidance for multivariable prediction models (see S2 Appendix) [33]. Ethical approval for each of the source studies was obtained from the relevant ethics committees in the country of data collection and from the trial sponsors (see S3 Appendix for list of ethics committees). All patients provided informed written consent.

## Results

### Baseline characteristics

**Fig 1:**
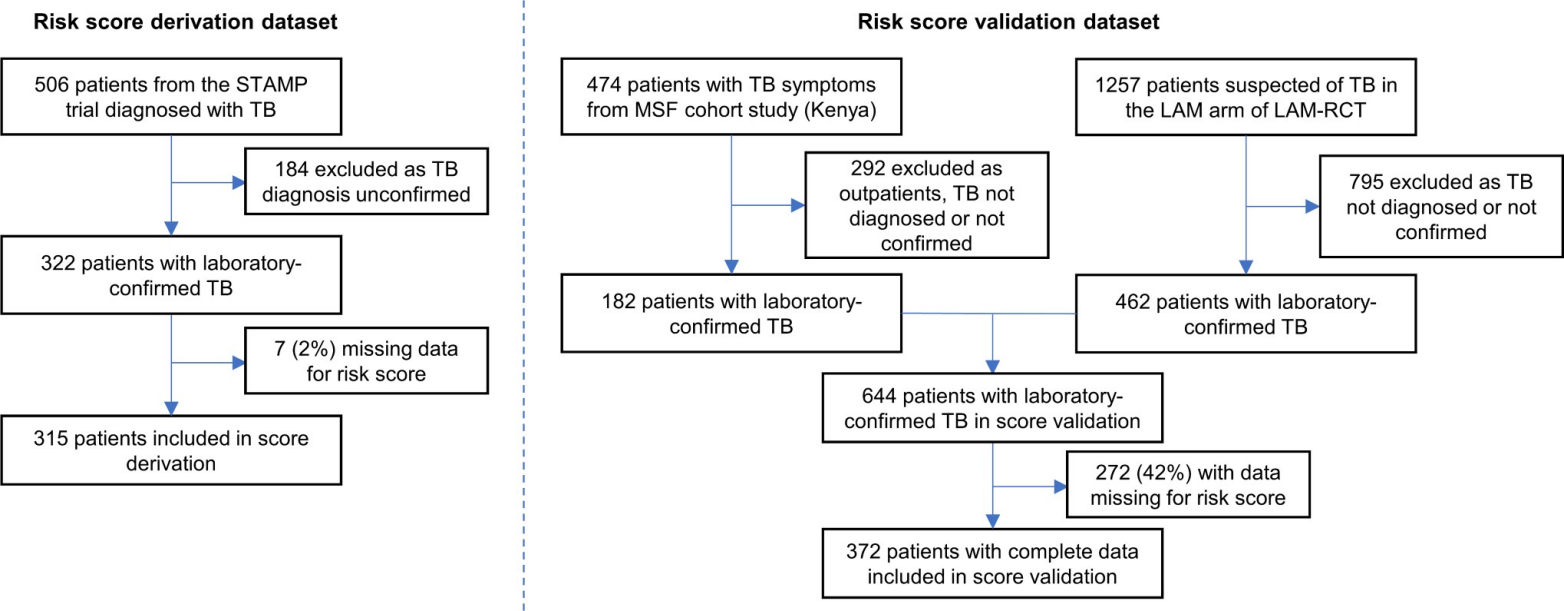
Study profile. LAM, lipoarabinomannan; MSF, Médecins Sans Frontières; TB, tuberculosis.

Of 506 HIV-positive patients diagnosed with TB in the STAMP trial derivation cohort, 322 had laboratory-confirmed TB. Seven patients were excluded from the complete case analysis for missing data (Fig 1). The median age of TB patients included in the derivation cohort was 36 years (interquartile range [IQR] 30–43), 172 (55%) were men, 53 (17%) were newly diagnosed with HIV, and the median CD4 cell count was 76 cells/μl (IQR 23–206; Table 1). In all, 209 (65%) patients were positive on urine TB-LAM testing, indicating probable disseminated TB disease. Anaemia was common and median haemoglobin was 86 g/l (IQR 67–108). Patients presented with advanced disease: 133 (42%) had 1 or more WHO danger signs, and 71 (23%) were severely disabled or unable to walk unaided.

**Table 1:** Baseline characteristics.

Characteristic	Category	Median (IQR) or *N* (%)	
		Derivation dataset (*n =* 315)	Validation dataset (*n =* 644)
**Demographics**			
Age (years)		36 (30–43)	35 (30–42)
Sex	Female	143 (45.4)	315 (48.9)
Country of enrolment	South Africa	162 (51.4)	102 (15.8)
	Tanzania	—	70 (10.9)
	Zambia	—	151 (23.5)
	Zimbabwe	—	139 (21.6)
	Kenya	—	182 (28.3)
	Malawi	153 (48.6)	—
**HIV history**			
New HIV diagnosis	Yes	53 (16.8)	—
Currently taking ART	Yes	210 (80.2)	290 (45.0)
Time on ART (years)^a^		1.0 (0.2–4.4)	0.7 (0.1–3.2)
CD4 cell count (cells/μl)^b^		76 (23–206)	61 (21–145)
**TB history**			
Cough	Yes	228 (72.4)	601 (93.5)
Fever	Yes	223 (70.8)	562 (87.3)
Weight loss	Yes	286 (90.8)	595 (95.8)
Night sweats	Yes	165 (52.4)	531 (82.6)
WHO TB symptom screen	Yes	310 (98.4)	644 (100)
Previous history of TB	Yes	72 (22.9)	123 (19.1)
**Clinical presentation**			
Weight (kg)^c^		50 (42–57)	49 (43–55)
BMI^d^		19.1 (16.2–21.0)	17.6 (15.9–20.3)
Heart rate (bpm)		104 (90–118)	102 (90–119)
Respiratory rate (per minute)		22 (20–26)	24 (22–28)
Systolic blood pressure (mm Hg)		102 (92–116)	104 (95–116)
Temperature (°C)		36.5 (36.1–37.2)	37.0 (36.6–38.0)
Haemoglobin (g/l)^e^		86 (67–108)	85 (68–100)
WHO danger sign^f^	Yes	133 (42.2)	399 (62.0)
Unable to walk unaided^g^	Yes	71 (22.5)	262 (40.7)
**TB diagnosis**			
Sputum Xpert positive	Yes	168 (52.2)	217 (33.7)
Sputum smear microscopy positive	Yes	—	211 (32.8)
TB culture positive (any site)	Yes	—	388 (60.3)
Urine LAM positive	Yes	209 (64.9)	424 (65.8)
Chest X-ray suggestive of TB	Yes	107 (33.2)	336 (52.2)
**Outcome**			
Died by 2 months	Yes	94 (29.8)	147 (22.8)

Sputum smear and TB culture were not routinely performed in the STAMP trial (derivation dataset). Missing data are for the validation dataset only.

^a^Time on ART missing for 19 (3%) patients.

^b^CD4 cell count missing for 27 (4%) patients.

^c^Weight missing for 75 (12%) patients.

^d^BMI missing for 90 (14%) patients.

^e^Haemoglobin missing for 272 (42%) patients.

^f^One of heart rate > 120 bpm, respiratory rate > 30 per minute, temperature > 39°C, or unable to walk unaided.

^g^Ability to walk unaided was assessed by healthcare worker and not self-reported.

ART, antiretroviral therapy; BMI, body mass index; bpm, beats per minute; IQR, interquartile range; LAM, lipoarabinomannan; TB, tuberculosis; WHO, World Health Organization; Xpert, Xpert MTB/RIF.

In the derivation cohort, 94 (30%) patients died within 2 months, with 66 (70%) dying during their hospital admission; 29 (31% of deaths) patients died by 1 week, and 52 (55%) by 2 weeks, after admission. In unadjusted analyses, mortality risk was higher in patients aged 55 years or older, men, ART-experienced patients, those unable to walk, patients with severe anaemia (haemoglobin < 80 g/l), patients with CD4 cell count < 100 cells/μl, and those with positive urine TB-LAM tests (Table 2). Six out of 322 (2%) patients were lost to follow-up after hospital discharge.

**Table 2:** Univariable and multivariable logistic regression analysis of factors associated with mortality in the derivation cohort (*n =* 315).

Characteristic	Category	Died^a^ (*n =* 94)	Univariable		Multivariable		Regression (β) coefficient
			OR (95% CI)	*p*-Value	OR (95% CI)	*p*-Value	
**Demographics**							
Age	<55 years	82 (28.4)	1 (ref)	0.067	1 (ref)		0.710
	≥55 years	12 (46.2)	2.2 (1.0–4.9)		2.0 (0.9–4.9)	0.10	
Sex	Female	32 (22.4)	1 (ref)	0.012	1 (ref)		0.923
	Male	62 (36.0)	2.0 (1.2–3.2)		2.5 (1.5–4.3)	0.001	
HIV infection							
ART experienced	No	18 (20.9)	1 (ref)	0.024	1 (ref)		0.621
	Yes	76 (33.2)	1.9 (1.1–3.4)		1.9 (1.0–3.5)	0.048	
CD4 cell count^#^	≥100 cells/μl	31 (23.9)	1 (ref)		—	—	—
	<100 cells/μl	62 (33.9)	1.7 (1.0–2.8)	0.040	—	—	—
Clinical presentation							
WHO danger sign	No	50 (27.5)	1 (ref)	0.185	—	—	—
	Yes	48 (34.8)	1.4 (0.9–2.2)		—	—	—
Weight	<35 kg	10 (43.5)	3.3 (1.2–8.9)	0.054	—	—	—
	35–60 kg	73 (31.5)	1.8 (0.9–3.5)		—	—	—
	>60 kg	11 (18.3)	1 (ref)		—	—	—
Haemoglobin (g/l)	≥80 g/l	44 (23.7)	1 (ref)	0.003	1 (ref)		0.703
	<80 g/l	50 (38.8)	2.0 (1.3–3.3)		2.0 (1.2–3.4)	0.008	
Unable to walk unaided	No	64 (26.2)	1 (ref)	0.004	1 (ref)		0.689
	Yes	30 (42.3)	2.2 (1.3–3.8)		2.0 (1.1–3.6)	0.022	
TB diagnosis							
Urine LAM positive	No	24 (22.6)	1 (ref)	0.044	1 (ref)		0.603
	Yes	70 (33.5)	1.7 (1.0–2.9)		1.8 (1.0–3.2)	0.040	

The constant (intercept) was −2.8. *p*-Values were calculated by likelihood ratio tests. There was no evidence of interaction between urine LAM positivity, being unable to walk, and haemoglobin < 80 g/l in the multivariable model (likelihood ratio test *p*-values all >0.1). Weight and being unable to walk were strongly associated.

^a^Data are number of patients in category who died (%).

ART, antiretroviral therapy; LAM, lipoarabinomannan; OR, odds ratio; TB, tuberculosis.

### Multivariable model and clinical risk score

The final multivariable logistic regression model for mortality at 2 months included age, sex, ART experience, haemoglobin, functional status (being unable to walk unaided), and urine TB-LAM result (Table 2). For associations of linear continuous variables with mortality see S1 Table. CD4 count and weight were dropped from the final predictor score model as their relationship with mortality was mediated by functional status and urine TB-LAM result. We found no significant interactions between variables in the final model. The c-statistic for the predictive model in the derivation dataset was 0.70 (95% CI 0.63–0.76), showing moderate discrimination. Calibration of the predictive model was good, as shown by the calibration plot (see S1 Fig) and a Hosmer-Lemeshow statistic *p =* 0.78.

**Fig 2:**
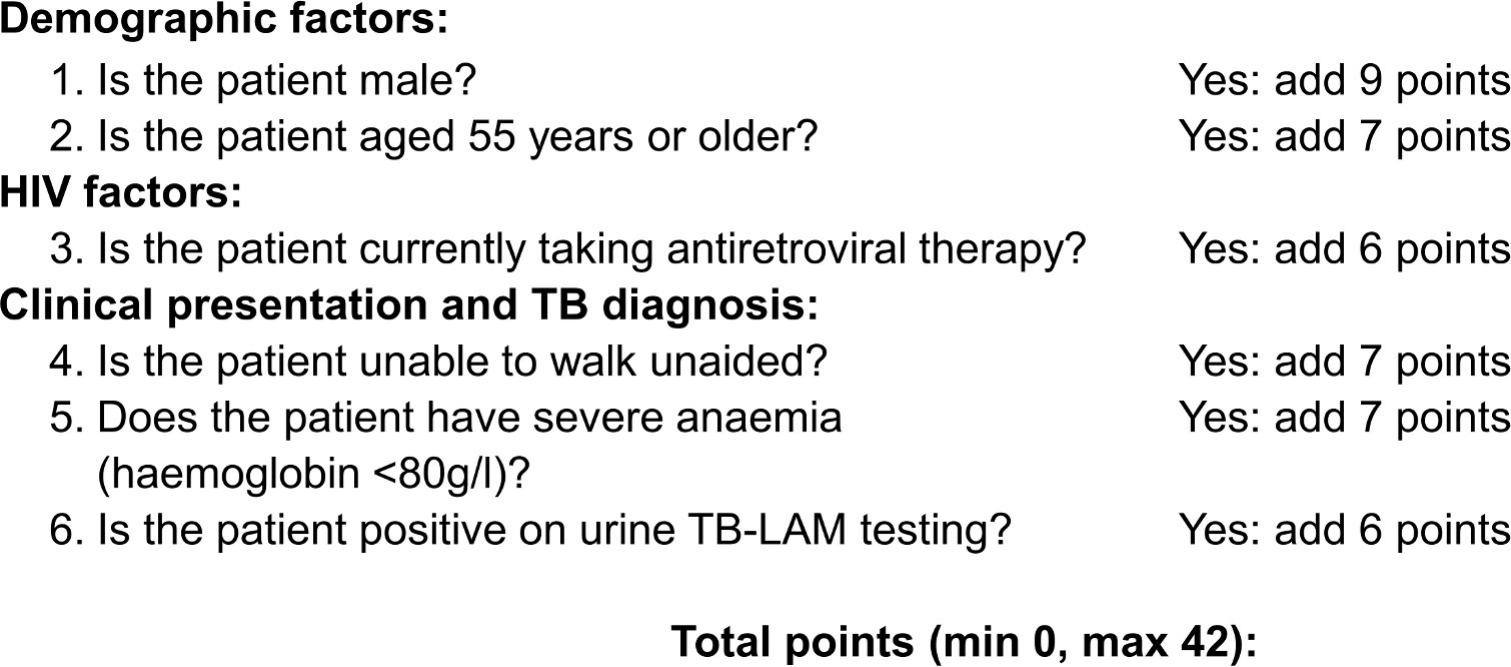
Risk score calculation to predict mortality. TB, tuberculosis; TB-LAM, Determine TB LAM Ag.

**Fig 3:**
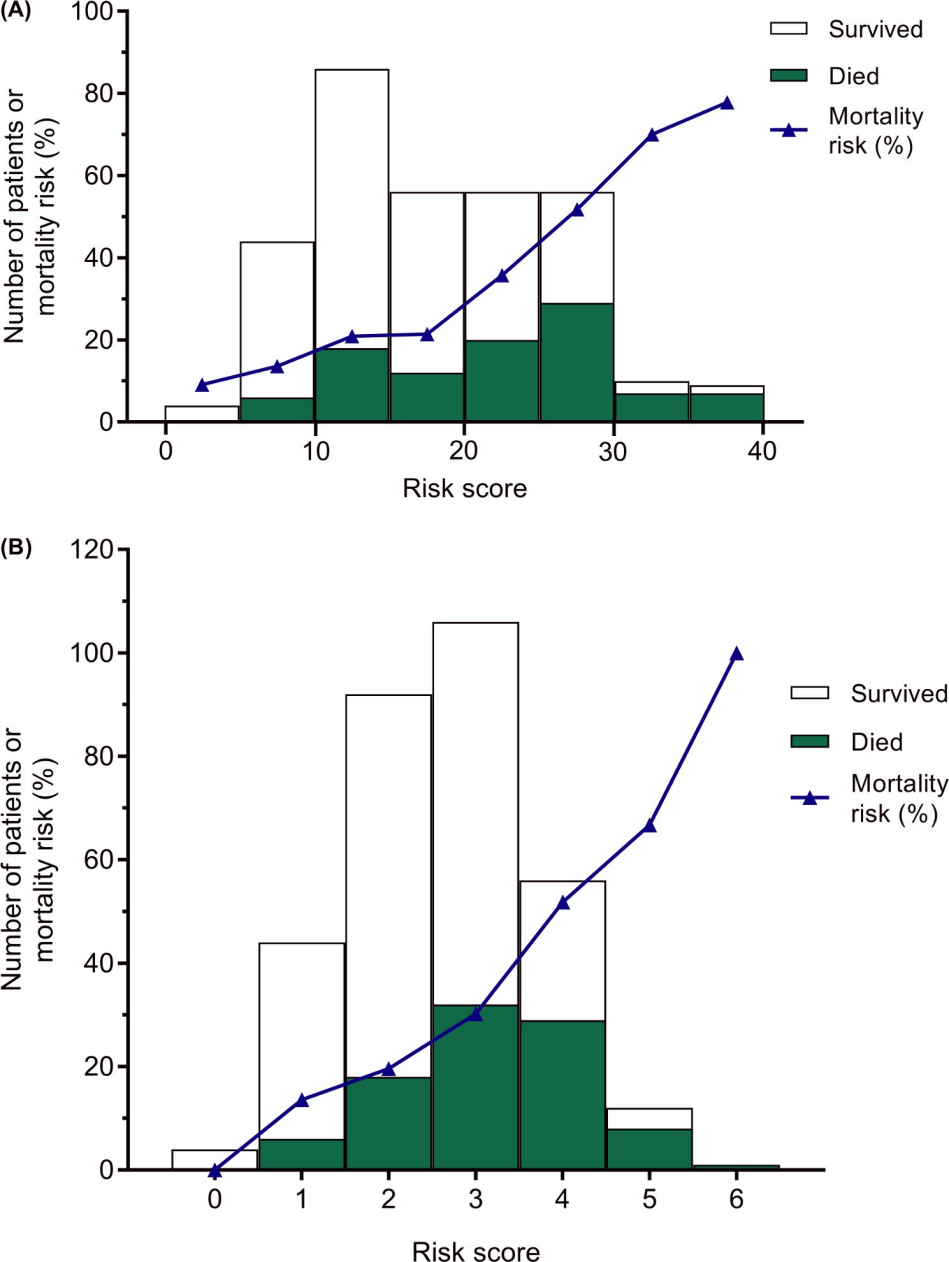
Distribution of risk scores and mortality in the derivation dataset. Distribution of risk scores for mortality stratified by outcome at 2 months (stacked bar chart) and mortality risk (percent, shown by blue line) for (A) the full risk score (based on the regression coefficients) and (B) the simplified risk score. Mortality risks and absolute numbers in each category are presented in S2 Table.

The clinical risk score for mortality, based on the regression coefficients, is outlined in Fig 2. Observed and predicted mortality risks for the risk score are reported in S2 Fig. Mortality risk groups were defined as low risk (10 points or fewer), medium risk (11 to 20 points), or high risk (more than 20 points) (Fig 3). Therefore, in the derivation cohort, 48 (15%) patients were deemed low risk, 142 (45%) were deemed medium risk, and 125 (40%) were deemed high risk. Median risk score was 19 (IQR 13–22, range 0–42). Observed mortality risk by 2 months was 12.5% (95% CI 5.7%–25.4%), 21.1% (95% CI 15.1%–28.7%), and 46.4% (95% CI 37.8%–55.2%) in the low-, medium-, and high-risk groups, respectively (*p <* 0.001). ORs for mortality were 6.1 (95% CI 2.4–15.2) in the high-risk group and 1.9 (95% CI 0.7–4.8) in the medium-risk group compared to low-risk patients (*p <* 0.001).

### Simplified clinical risk score for mortality

As the regression coefficients and points in the clinical risk score were similar for all 6 variables, we created a simplified version of the score by assigning each variable within the score 1 point if present (age 55 years or over, male sex, ART experienced, severe anaemia, being unable to walk unaided, or urine TB-LAM positive; see S3 Fig). A high mortality risk was defined as 3 or more points, medium risk as 2 points, and low risk as 0 or 1 point.

In the derivation cohort, patients with 3 or more points (high risk) had a mortality of 40.0% (70/175, 95% CI 33.0%–47.5%), compared to 19.6% (18/92, 95% CI 12.6%–29.0%) mortality in those with 2 points and 12.5% (6/48, 95% CI 5.7%–25.4%) in those with 0 or 1 point (*p <* 0.001) (see Fig 3 and S2 Table). The sensitivity of the risk score for mortality in was 0.75 (the score correctly identified 70/94 deaths), and the positive predictive value was 0.4.

**Fig 4:**
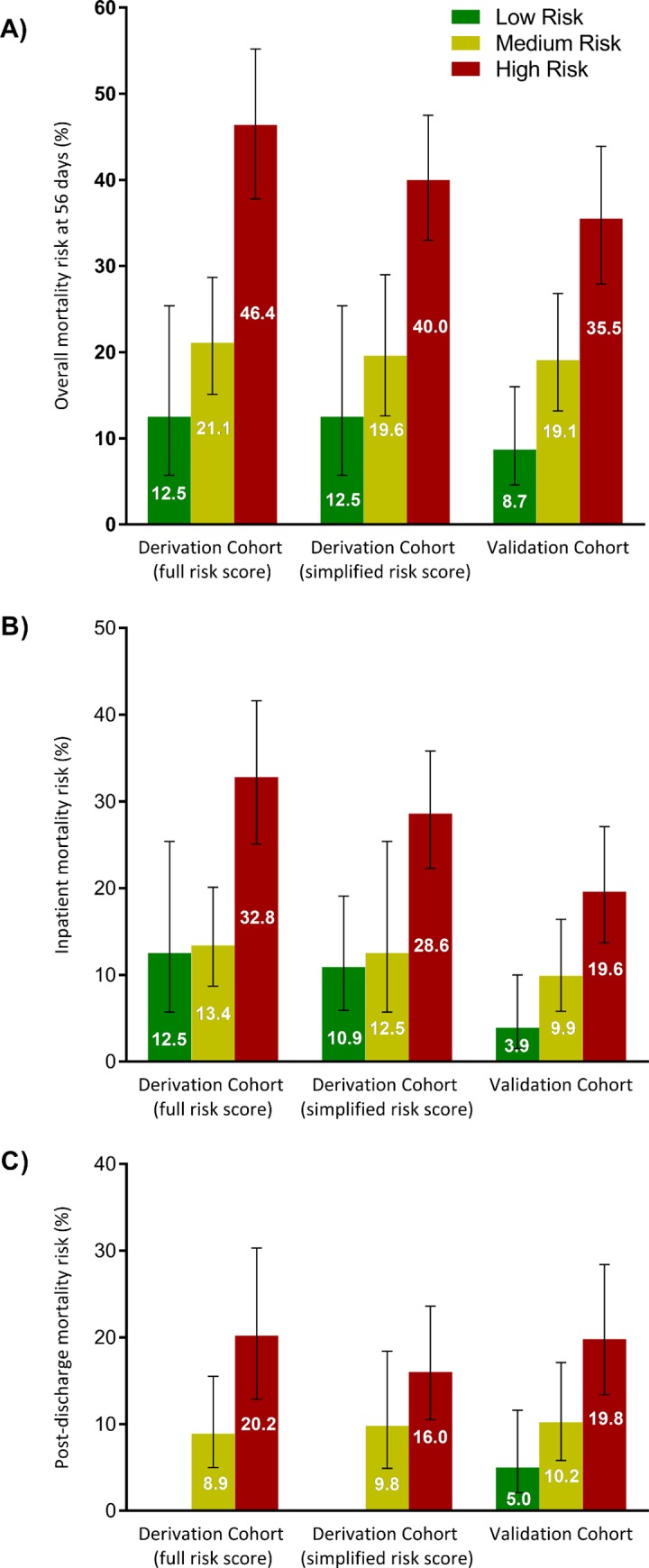
Observed mortality risk by risk score category in the derivation and validation cohorts. Observed mortality risk (A) at 56 days, (B) during inpatient stay, and (C) post-discharge in the derivation and validation cohorts, stratified by risk score category (derivation and validation cohorts) and simplified risk score category (derivation cohort). Numbers on bars represent absolute mortality risk; error bars represent 95% confidence intervals. For the full risk score, low risk was defined as 10 points or fewer, medium risk as 11 to 20 points, and high risk as more than 20 points. For the simplified risk score, the low-risk group had a predictor score of 0 or 1 point, the medium-risk group had a predictor score of 2 points, and the high-risk group had predictor score of ≥3 points. *p-*Values based on chi-squared tests between groups for derivation and validation cohorts, respectively, are (A) *p <* 0.001 and *p <* 0.001, (B) *p =* 0.001 and *p =* 0.001, and (C) *p =* 0.015 and *p =* 0.003.

**Fig 5:**
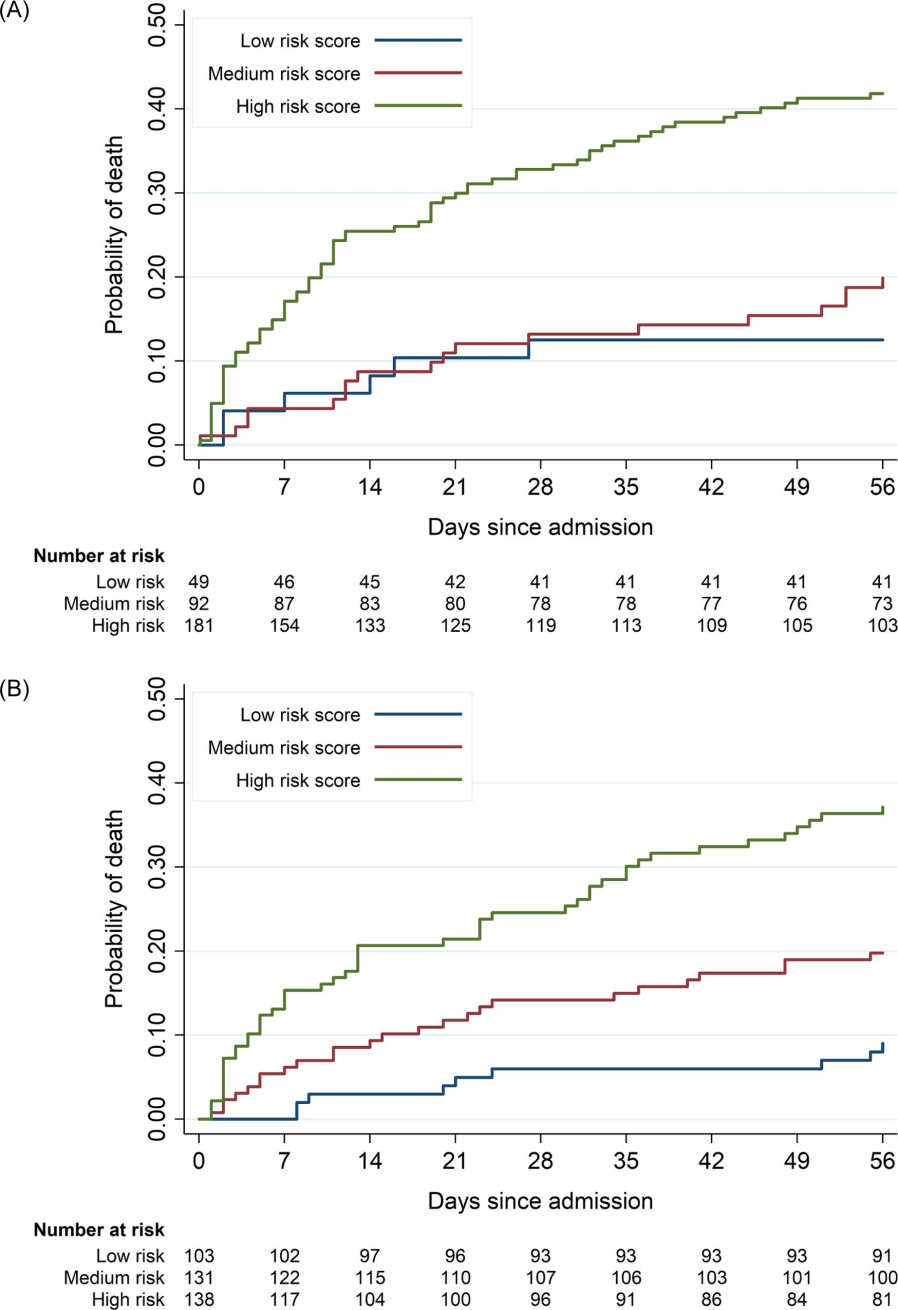
Survival curves stratified by clinical risk score category in the derivation dataset. Survival curves and risk tables with number at risk for the (A) derivation cohort and (B) validation cohort stratified by risk group using the simplified clinical risk score. The low-risk group (blue line) had a risk score of 0 or 1 point, the medium-risk group (red line) had a risk score of 2 points, and the high-risk group (green line) had a risk score of ≥3 points. Log-rank test *p <* 0.001.

The clinical risk score was useful in predicting deaths that occurred during inpatient admission (50 [28.6%, 95% CI 22.3%–35.8%] in the high-risk group compared to 6 [10.9%, 95% CI 5.9%–19.1%] in the low-risk group, *p =* 0.001) as well as deaths occurring after discharge (20 [16.0%, 95% CI 10.5%–23.6%] in the high-risk group compared to 0 [0%] in the low-risk group, *p =* 0.015; Fig 4). More patients in the high-risk group were TB-LAM positive and had higher grades of positive result, but CD4 cell count did not differ by risk group (see S4 Fig). Survival curves by risk group are presented in Fig 5.

### External validation

The external validation cohort included 644 HIV-positive patients with laboratory-confirmed TB, of whom 372 (58%) patients had no missing data for the risk score and were therefore included in the complete case analysis (Fig 1). Baseline characteristics were similar between cohorts, although fewer patients reported taking ART and more patients presented with severe functional impairment and 1 or more WHO danger signs in the validation cohort (Table 1). A similar proportion of patients were positive on urine TB-LAM testing (65% in the derivation cohort compared to 66% in the validation cohort). Mortality at 2 months was lower in the validation cohort (22.8%) compared to the derivation cohort (29.8%). Loss to follow-up was 4% in the validation cohort (15/372).

In complete case analysis (*n =* 372), the observed mortality risks in the validation cohort were 8.7% (95% CI 4.6%–16.0%) in the low-risk group, 19.1% (95% CI 13.2%–26.8%) in the medium-risk group, and 35.5% (95% CI 27.9%–43.9%) in the high-risk group (see Fig 4). Median risk score was 16 (IQR 10–22, range 0–42). The ORs for mortality by risk group were similar to those in the derivation cohort (5.8 [95% CI 2.7–12.3] for the high-risk group and 2.5 [95% CI 1.1–5.5] for the medium-risk group compared to the low-risk group). The risk score was also useful in predicting both inpatient and post-discharge deaths (the high-risk group had a 20% risk of post-discharge death compared to 5% in the low-risk group). The simplified risk score performed similarly to the full score in the validation cohort.

The predictive model had similar calibration and discrimination in the validation cohort as in the derivation cohort: the c-statistic was 0.68 (95% CI 0.61–0.74; see S3 Table), and the Hosmer-Lemeshow statistic had *p =* 0.13 (see S5 Fig for the calibration plot). In a sensitivity analysis using multiple imputation for missing data in the validation dataset (*n =* 644), the c-statistic for the predictive model was 0.64 (95% CI 0.60–0.69), and the Hosmer-Lemeshow statistic had *p =* 0.67. ORs for mortality were 5.3 (95% CI 2.2–9.5) for the high-risk group and 2.1 (95% CI 1.0–4.6) for the medium-risk group compared to low-risk patients (*p* < 0.001).

## Discussion

In this study, we developed and externally validated a pragmatic clinical risk score to predict early mortality in HIV-positive patients admitted to hospital and diagnosed with laboratory-confirmed TB. Our score used 6 clinical and laboratory factors that could be readily collected at admission to hospital in settings with high HIV and TB burden. The score was able to categorise patients into 3 risk groups. One-third of the high-risk group died during hospital admission, and almost 50% had died by 2 months. A simplified ‘3 of 6 predictors’ version of the score performed similarly. This is the first study to our knowledge to derive and externally validate a risk score to predict mortality in this patient population.

We found older age, being male, being ART experienced, having severe anaemia, being severely disabled or unable to walk unaided, and being urine TB-LAM positive were all risk factors for mortality. These factors have been established as being associated with outcome in HIV/TB in previous studies [24–28], and most likely reflect more advanced HIV-related immunosuppression, late presentation to healthcare services, and/or poorer underlying physiological reserve. Positive urine diagnostic tests (including LAM detection and *Mycobacterium tuberculosis* nucleic acid detection) in the context of HIV infection are thought to represent haematogenously disseminated renal TB with high mycobacterial burden, which may explain why it is associated with a worse prognosis [34]. Interestingly, clinical signs and symptoms (such as WHO danger signs) were not predictive of mortality.

In contrast to previously published data, ART-experienced patients had a higher mortality risk in our study [28,35]. This likely reflects a high burden of unrecognised ART failure, due to either poor adherence or drug resistance among patients admitted to hospital. Another potential cause is immune reconstitution inflammatory syndrome (IRIS) in patients who have recently started ART. The relationship between ART and mortality is likely to be more complex, representing different groups of patients with different mortality risks, but for this pragmatic tool we have not been able to explore this further. CD4 cell count, which has been previously shown to be associated with mortality in HIV-positive patients, dropped out of our final multivariable predictive model due to mediation by other variables. Furthermore, in the era of test and treat for HIV and use of quantitative HIV viral load for monitoring, CD4 testing services are being scaled back, and are often not available in resource-limited settings.

Mechanisms and causes of mortality in advanced HIV/TB are still not well understood. Co-pathologies, including other opportunistic infections and bacterial pneumonia or sepsis, are commonly detected post-mortem [36,37]. High-risk patients could be prioritised for screening for co-infections, for example using cryptococcal antigen point-of-care tests, or empirical prophylactic treatment with antibacterial agents, an approach that has been shown to reduce mortality in advanced HIV infection [38].

Whilst this clinical risk score can identify patients with the highest risk of mortality, there remains an absence of proven interventions (beyond TB therapy and appropriately timed ART) to reduce mortality in this population. Therefore, we propose this score could be used as a clinical tool to alert clinicians to patients at high risk of mortality who should be reviewed before discharge and/or flagged for early clinical follow-up in settings where urine TB-LAM scale-up is occurring. The score could also be used as a research tool to aid evaluation of intensified or optimised TB treatment regimens or adjunctive interventions aimed at reducing high mortality in this population.

Possible interventions include rapid viral load testing with ART adherence support and early switching for those with virological failure. Host-directed therapies, which target host immune responses, are in clinical trials for TB, including some specifically for HIV/TB [39,40]. Patients identified as being at highest risk for mortality could also be offered more intensive monitoring or supportive care, for example better management of severe anaemia [41], although optimal strategies of supportive care are not clear [42]. Enhanced treatment and prophylaxis for co-infections have been shown to reduce early mortality in patients with advanced HIV initiating ART [38], and may also benefit those with HIV/TB disease. Interventions will likely need to be instituted rapidly after TB diagnosis to alter outcomes.

The risk score was able to highlight patients at highest risk of death post-discharge, in addition to those at high risk of death during hospitalisation, and could be used to prevent too early discharges. Enhanced community support, including home visits, has been shown to reduce mortality after starting ART in advanced HIV [43], and could have a similar impact for HIV/TB patients. Current services in high-burden settings take a public health approach to service delivery, whereas prognostic risk scores can identify patients suitable for differentiated care [8].

The main aim of this risk score was to detect patients at high risk of early mortality who may benefit from interventions in addition to TB treatment. Although the discrimination of the model was not perfect, the sensitivity of the simplified score was 75%; the score did not identify 25% of patients who died within 2 months, and such patients would still receive standard-of-care management of HIV/TB. Proposed interventions to reduce mortality would have limited adverse events, so those deemed as ‘high risk’ by the score but surviving to 2 months are unlikely to come to significant harm from such interventions. However, if adjunctive interventions are found to reduce early mortality, better predictive biomarkers or more accurate predictive tools would allow more efficient use of resources through targeting of patients.

Limitations of our study include the potential for selection bias. In the STAMP trial standard-of-care arm, only patients started on TB treatment for clinical/radiological criteria or following a positive sputum Xpert result had stored urine retrieved for TB testing. Patients with otherwise undiagnosed TB who would have been urine test positive if they had been tested were not included in this study. Patients unable to provide consent, mostly due to being severely unwell and having altered consciousness, were also excluded. Although our risk score did not have optimal discrimination and calibration, performance was adequate and similar to that of other prognostic scores widely used in clinical practice (e.g., the Framingham cardiovascular risk score) [15,44]. Performance may have been reduced by categorising continuous variables for simplicity. TB drug resistance was not a predictor of mortality in this cohort; however, prevalence of rifampicin resistance was low in these settings. Not all established risk factors for mortality were characterised, leaving potential to improve on performance. Future studies could assess more detailed markers of physiology, as well as social and more distal risk factors.

Whilst the score is pragmatic and its constituent factors are widely available in hospitals in African regions with high HIV and TB burdens, it does rely on access to the TB-LAM lateral flow assay. There is now good evidence to support mortality reductions with the use of TB-LAM in HIV-positive patients admitted to hospital [4,5], and its use as a screening test has been incorporated into the latest guidelines in Malawi and South Africa. The assay has also been scaled up nationally in eSwatini, Kenya, and Uganda [45]. Missing data were common in the validation cohort. However, sensitivity analyses using multiple imputation gave similar results as the complete case analysis. We assumed patients lost to follow-up were alive at 2 months, although only 2% in the derivation cohort and 4% in the validation cohort were not followed up after hospital discharge. Our cohort did not include patients treated for TB without a positive diagnostic test, which remains common in HIV-positive patients admitted to hospital, and this patient group may be an important group for whom to apply risk stratification and predictive scores. The biomarkers studied are imperfect predictors of mortality, and further research is needed to focus on better biomarkers to predict outcome.

Strengths of this study include that the derivation cohort and the LAM-RCT external validation cohort were nested within randomised controlled trials. Our predictive model had similar discrimination and calibration in the validation cohort, and was able to identify groups of patients with similarly increased odds of mortality. This was despite the validation cohort being from geographically distinct locations, collected at different times by different investigators, and with a lower overall mortality risk at 2 months. The factors required for the score can be obtained rapidly after admission.

In conclusion, we have developed and externally validated a clinical risk score capable of identifying, among patients admitted to hospital in settings with high HIV/TB burden, those with the highest risk of early mortality. This score could be a useful clinical and research tool, and could prove beneficial in identifying patients who would gain most from adjunctive interventions to reduce mortality. Further work to assess the impact of such risk scores, and to identify which interventions could potentially reduce mortality, is urgently needed if ambitious global targets to reduce TB mortality are to be met by 2025.

## Supporting information

S1 Appendix Statistical analysis plan. Prospective statistical analysis plan (version 1.2, 4 June 2017).
(PDF)

S2 Appendix TRIPOD checklist. (PDF)

S3 Appendix List of ethics committees that provided approval. (PDF)

S1 Fig Performance of clinical risk score in derivation cohort. (A) Receiver operator curve of the predictive model: area under the curve = 0.70 (95% CI 0.63–0.76). (B) Calibration plot of observed probability of mortality plotted against predicted probability of mortality by the risk score multivariable regression model, with variables grouped into deciles based on predicted probability, and 95% CIs. Black dashed line shows perfect prediction. Hosmer-Lemeshow statistic *p =* 0.78.
(TIF)

S2 Fig Observed and predicted mortality for risk score values in the derivation cohort. The size of the blue circles representing observed mortality risk is proportional to the number of patients with that score. Predicted mortality risk is represented by the green line/triangles.
(TIF)

S3 Fig Risk score calculation for the simplified risk score to predict mortality. (TIF)

S4 Fig Urine TB-LAM grade and CD4 cell strata stratified by clinical risk score for the derivation cohort (*n =* 315). (TIF)

S5 Fig Calibration plot for the predictive model in the external validation dataset (*n =* 372). Plot shows the observed compared to expected probability of risk for the external validation cohort as deciles based on risk score, with 95% CIs. Hosmer-Lemeshow statistic *p =* 0.13. c-Statistic (or area under the receiver operator curve) was 0.68 (95% CI 0.61–0.74). Dotted line represents perfect prediction.
(TIF)

S1 Table Univariable analysis of continuous variables and associations with mortality. (PDF)

S2 Table Risk score and mortality data. Number of patients surviving and patients dying, and observed and predicted mortality risk for (A) the full clinical risk score (based on the regression coefficients) and (B) the simplified risk score.
(PDF)

S3 Table C-statistic, 95% confidence intervals, and Hosmer-Lemeshow test for final model and risk scores in derivation and validation cohorts. (PDF)

## Abbreviations

ART: antiretroviral therapy
C-index: concordance index
HIV/TB: HIV-associated tuberculosis
LAM: lipoarabinomannan
MSF: Médecins Sans Frontières
OR: odds ratio
TB: tuberculosis
TB-LAM: Determine TB LAM Ag
Xpert: Xpert MTB/RIF
