# Supplementary material for: Risk score for predicting mortality including urine lipoarabinomannan detection in hospital inpatients with HIV-associated tuberculosis in sub-Saharan Africa: Derivation and external validation cohort study
Source: PLoS Med. 2019 Apr 5;16(4):e1002776. doi: 10.1371/journal.pmed.1002776 (PMC6450614; doi:10.1371/journal.pmed.1002776)
Supplement: S1 Appendix — Prospective statistical analysis plan (version 1.2, 4 June 2017). (PDF) [file pmed.1002776.s001.pdf]

High mortality amongst hospitalised patients with HIV-associated TB despite TB treatment: derivation and validation of a predictive tool

Statistical Analysis Plan

Introduction and background

Despite the initiation of TB treatment and early ART, mortality in patients with HIV-associated TB (HIV-TB) who are admitted to hospital remains high.[1] A recent systematic review and meta-analysis found a 29% (95% CI 20-38%) mortality among hospitalised adults with HIV-TB.[2] Predictors of mortality vary between studies, but include current CD4 cell count, anaemia, indices of malnutrition and, as recently shown in a systematic review, urinary LAM-detection.[3–14]

Clinical predictor and prognostic scores have been developed and validated for HIV-positive patients presenting with PCP,[15] Cryptococcal meningitis [16] and pneumonia.[17] The TBScore was developed to predict outcome in pulmonary TB,[18] and a score to predict MTB bacteraemia amongst HIV-positive hospital admissions has also been developed.[19] No tool to predict mortality in HIV-TB patients admitted to hospital in sub-Saharan Africa (SSA) has been developed. Furthermore, the aforementioned prediction tools have not been externally validated in different populations or settings, or had their impact evaluated.

A prognostic scoring tool for mortality in HIV-TB could be used in clinical practice to enhance the clinical care of patients with the worst prognosis. It could also have research applications, including aiding the development and evaluation of adjunctive interventions for HIV-TB, and identifying those patients who may benefit from such interventions.

Aim

To develop and validate a pragmatic, clinically applicable tool for use at admission for predicting early mortality (defined as < 2 months) in patients with HIV-TB admitted to hospital in SSA.

Study population

The population for developing the tool will be patients who are HIV-positive who are admitted to hospital and diagnosed with microbiologically/laboratory confirmed TB, defined as any positive:

- Mycobacterial culture

- Xpert MTB/RIF assay
- Determine TB-LAM assay

Outcome

The outcome for this study will be early mortality, defined as death within 2 months of admission.

Methods: developing the prognostic model

A cohort of patients with microbiologically confirmed HIV-TB admitted to hospital in high-prevalence settings in sub-Saharan Africa nested within the STAMP clinical [ref] trial will be used to develop the prognostic model.

- Clinically relevant and pragmatic demographic and clinical variables will be chosen for possible inclusion in the model based upon (1) a priori knowledge from existing studies, (2) based on univariate associations with mortality in development dataset (3) the need for unambiguous, reproducible variables that are available in most clinical settings where the tool may be used.
- In keeping with good practice, a minimum of 10 events (deaths) will be required for each candidate predictor studied.[25]
- Continuous variables (such as CD4 cell count and haemoglobin) will be kept as continuous predictors when practical or converted to ordered categorical variables or dichotomised to simplify the score (based on previously established cut-offs or data-derived categories based on associations with outcomes). Continuous variables will be assessed for non-linearity using fractional polynomials functions, and may need to be transformed in the regression models.
- Univariate associations between demographic and clinical variables and mortality will be tested using χ^2 and risk ratios with 95% Cis and/or logistic regression.
- A backward elimination stepwise approach to logistic regression modelling will be used to identify candidate predictor variables. Variables will be kept in the model based on p-values using likelihood ratio tests and Akaike information criterion (AIC), aiming to avoid overfitting.
- Further evaluation will include testing for interaction and comparing transformations of predictors where appropriate
- A clinical predictor score will be developed by giving dichotomous variables a value of 0 or 1, and using the regression coefficients from the logistic regression model to calculate the relative contribution of the variable to the risk score. Regression co-efficients will be

rounded to integers and/or scaled ans assigned as points to each variable in the final risk score

- The score will be a continuous factor and an ordered categorical factor for simplification, with risk score categories being created based on plotting risk score against observed mortality.
- Missing data will be assumed to be missing at random. Complete case analysis will be used if observations missing data are <5%, otherwise multivariable multiple imputation with chained equations will be used to deal with missing data.

Methods: validating the prognostic model

The performance of the model will be evaluated on:

- Model discrimination (ability to differentiate patients who would die within 2 months to those who survived) by calculating the concordance (C)-index (also known as the area under the receiver operator curve), assuming a C-index <0.6 showed poor discrimination [26]
- Model calibration, assessed by plotting the probability of mortality predicted by the model against those observed in the derivation dataset using a calibration plot and the Hosmer-Lemeshow test, assuming a $p<0.05$ indicated poor calibration
- Potential clinical usability, practicality and face validity.
- Although the primary outcome measure is mortality risk at 2-months, exploratory analyses will evaluate the performance of the predictive score for deaths at different times by stratifying deaths as early or late (this will be defined based on the distribution of time to death)

Internal validation of the model will be done using bootstrapping of the development dataset to stimulate 100 'resampled' populations of the same size.

External validation of the model will be done using recent (2012 or later) hospital cohorts of HIV-TB patients from the high-prevalence HIV and TB countries in sub-Saharan Africa. Data is from MSF Homa Bay cohort study and LAM RCT clinical trial) is available and planned for external validation.

- Performance of the model will be based on both calibration and discrimination
- Calibration will be investigated by comparing expected mortality outcomes based on the predictive model and actual outcomes and compared using χ^2test. The values will also be plotted for comparison, and accompanied by the Hosmer-Lemeshow 'goodness of fit' test

- Discrimination will be tested using both area under the receiver operating curve (the concordance index/C-statistic).
- Mortality risks will be calculated based on the risk score (both either a continuous and/or ordered categorical variable as defined for the derivation cohort)

If performance of the model is sub-optimal, it may be adjusted (either re-calibrated or revised) using the data in the validation dataset. This should improve stability and generalisability of the model.

Impact of the prognostic model

Finally, the outline of studies to test the impact of the clinical prediction tool will be developed and presented.

Ethical considerations

As this study is using secondary data, ethical approval will not be required. However, the original studies in which the data was collected should have undergone approval from the appropriate ethical committee(s)/review boards.

References

1. World Health Organization: *Global Tuberculosis Report 2016*. Geneva; 2016.

2. Ford N, Matteelli A, Shubber Z, Hermans S, Meintjes G, Grinsztejn B, Waldrop G, Kranzer K, Doherty M, Getahun H: **TB as a cause of hospitalization and in-hospital mortality among people living with HIV worldwide : a systematic review and meta-analysis**. *J Int AIDS Soc* 2016, **19**:20714.

3. Talbot E, Munseri P, Teixeira P, Matee M, Bakari M, Lahey T, von Reyn F: **Test characteristics of urinary lipoarabinomannan and predictors of mortality among hospitalized HIV-infected tuberculosis suspects in Tanzania**. *PLoS One* 2012, **7**:1–6.

4. Manabe YC, Nonyane BAS, Nakiyingi L, Mbabazi O, Lubega G, Shah M, Moulton LH, Joloba M, Ellner J, Dorman SE: **Point-of-care lateral flow assays for tuberculosis and cryptococcal antigenuria predict death in HIV infected adults in Uganda**. *PLoS One* 2014, **9**:11–14.

5. Diendéré EA, Badoum G, Bognounou R, Guira O, Ilboudo L, Tieno H, Diallo I, Drabo J: **Clinical outcomes and mortality associated factors in patients infected with HIV receiving a presumptive anti-tuberculosis treatment in a tertiary level hospital in Burkina Faso.** *AIDS Care* 2015, **27**:1250–4.

6. Kirenga BJ, Levin J, Ayakaka I, Worodria W, Reilly N, Mumbowa F, Nabanjja H, Nyakoojo G, Fennelly K, Nakubulwa S, Joloba M, Okwera A, Eisenach KD, McNerney R, Elliott AM, Mugerwa RD, Smith PG, Ellner JJ, Jones-López EC: **Treatment outcomes of new tuberculosis patients hospitalized in Kampala, Uganda: A prospective cohort study**. *PLoS One* 2014, **9**:1–10.

7. Subbarao S, Wilkinson KA, van Halsema CL, Rao SS, Boyles T, Utay NS, Wilkinson RJ, Meintjes G: **Raised Venous Lactate and Markers of Intestinal Translocation Are Associated With Mortality Among In-Patients With HIV-Associated TB in Rural South Africa.** *J Acquir Immune Defic Syndr* 2015, **70**:406–13.

8. Holtz TH, Kabera G, Mthiyane T, Zingoni T, Nadesan S, Ross D, Allen J, Chideya S, Sunpath H, Rustomjee R: **Use of a WHO-recommended algorithm to reduce mortality in seriously ill patients with HIV infection and smear-negative pulmonary tuberculosis in South Africa: An observational cohort study**. *Lancet Infect Dis* 2011, **11**:533–540.

9. CA B, EM I, JJ T, CF K: **Tuberculosis in the intensive care unit: a prospective observational study.** *Int J Tuberc Lung Dis* 2014, **18**:824–830.

10. Ravimohan S, Tamuhla N, Steenhoff AP, Letlhogile R, Nfanyana K, Bellamy SL, MacGregor RR, Gross R, Weissman D, Bisson GP: **Immunological profiling of tuberculosis-associated immune reconstitution inflammatory syndrome and non-immune reconstitution inflammatory syndrome death in HIV-infected adults with pulmonary tuberculosis starting antiretroviral therapy: a prospective obse**. *Lancet Infect Dis* 2015, **15**:429–438.

11. Bigna JJR, Noubiap JJN, Agbor AA, Plottel CS, Billong SC, Ayong APR, Koulla-Shiro S: **Early mortality during initial treatment of tuberculosis in patients co-infected with HIV at the Yaound?? Central Hospital, Cameroon: An 8-year retrospective cohort study (2006-2013)**. *PLoS One* 2015, **10**:1–13.

12. Kyeyune R, den Boon S, Cattamanchi A, Davis JL, Worodria W, Yoo SD, Huang L, *Makerere: **Causes of Early Mortality in HIV-Infected TB Suspects in an East African Referral Hospital**. *J Acquir Immune Defic Syndr* 2010, **55**:446–450.

13. Marcy O, Laureillard D, Madec Y, Chan S, Mayaud C, Borand L, Prak N, Kim C, Lak KK, Hak C, Dim B, Sok T, Delfraissy J-F, Goldfeld AE, Blanc F-X: **Causes and determinants of mortality in HIV-infected adults with tuberculosis: an analysis from the CAMELIA ANRS 1295-CIPRA KH001 randomized trial.** *Clin Infect Dis* 2014, **59**:435–45.

14. Crump J a, Ramadhani HO, Morrissey AB, Saganda W, Mwako MS, Yang L-Y, Chow S-C, Njau BN, Mushi GS, Maro VP, Reller LB, Bartlett J a: **Bacteremic disseminated tuberculosis in sub-saharan Africa: a prospective cohort study.** *Clin Infect Dis* 2012, **55**:242–50.

15. Armstrong-James D, Copas a J, Walzer PD, Edwards SG, Miller RF: **A prognostic scoring tool for identification of patients at high and low risk of death from HIV-associated Pneumocystis jirovecii**

pneumonia. *Int J STD AIDS* 2011, **22**:628–34.

16. Jarvis JN, Bicanic T, Loyse A, Namarika D, Jackson A, Nussbaum JC, Longley N, Muzoora C, Phulusa J, Taseera K, Kanyembe C, Wilson D, Hosseinipour MC, Brouwer AE, Limmathurotsakul D, White N, Van Der Horst C, Wood R, Meintjes G, Bradley J, Jaffar S, Harrison T: **Determinants of mortality in a combined cohort of 501 patients with HIV-associated cryptococcal meningitis: Implications for improving outcomes**. *Clin Infect Dis* 2014, **58**:736–745.

17. Koss CA, Jarlsberg LG, Den Boon S, Cattamanchi A, Davis JL, Worodria W, Ayakaka I, Sanyu I, Huang L, Davis L, Andama A, Byanyima P, Kalema N, Katagira W, Awor M, Kaswabuli S, Nabakiibi C: **A clinical predictor score for 30-day mortality among HIV-infected adults hospitalized with pneumonia in Uganda**. *PLoS One* 2015, **10**:1–12.

18. Rudolf F, Lemvik G, Abate E, Verkuilen J, Schön T, Gomes VF, Eugen-Olsen J, Østergaard L, Wejse C: **TBscore II: refining and validating a simple clinical score for treatment monitoring of patients with pulmonary tuberculosis.** *Scand J Infect Dis* 2013, **45**(April):825–36.

19. Jacob ST, Pavlinac PB, Nakiyingi L, Banura P, Baeten JM, Morgan K, Magaret A, Manabe Y, Reynolds SJ, Liles WC, Wald A, Joloba ML, Mayanja-Kizza H, Scheld WM: **Mycobacterium tuberculosis bacteremia in a cohort of hiv-infected patients hospitalized with severe sepsis in uganda–high frequency, low clinical suspicion [corrected] and derivation of a clinical prediction score.** *PLoS One* 2013, **8**:e70305.

Appendix 1: Candidate variables for development dataset

Category	Variable	Comment
**Demographics**	Age	
	Gender	
	Date of admission	
	Site/study	
**HIV**	New diagnosis	Yes/No
	ART status	Never/Currently On/Interrupted etc..
	Time on ART/Date ART started	
**TB**	Previous history of TB	Yes/No
	Clinically suggested TB	Yes/No
	Date of starting TB treatment	
	Reason for starting TB treatment	
**Admission**	Reason for admission	
	Duration of illness	
	Presence of TB symptoms	Cough, fever, night sweats, weight loss
	WHO TB symptom screen	
	Respiratory rate	
	Heart rate	
	Systolic BP	
	Temperature	
	Karnofsky score/assessment of function	
	WHO danger signs	
	Weight/BMI	
**Lab results (at admission)**	CD4 cell count (absolute)	
	Haemoglobin	
	C-Reactive Protein	
**TB diagnostics**	Urine LAM	Positive/Negative/Not done
	Urine LAM grade	1,2,3,4,5
	Sputum Xpert	Positive/Negative/Not done
	Non-sputum Xpert	Positive/Negative/Not done
	Sputum culture	Positive/Negative/Not done
	Non-sputum culture	Positive/Negative/Not done
	CXR suggestive of TB	Yes/No/Not done
**Outcome**	All-cause 2 month mortality	Yes/No/LTFU
	Date of death	
	Date of discharge	
