# Supplementary material for: Risk score for predicting mortality including urine lipoarabinomannan detection in hospital inpatients with HIV-associated tuberculosis in sub-Saharan Africa: Derivation and external validation cohort study
Source: PLoS Med. 2019 Apr 5;16(4):e1002776. doi: 10.1371/journal.pmed.1002776 (PMC6450614; doi:10.1371/journal.pmed.1002776)
Supplement: S2 Appendix — (PDF) [file pmed.1002776.s002.pdf]

TRIPOD Checklist: Prediction Model Development and Validation

Section/Topic	Item		Checklist Item	Page
**Title and abstract**				
Title	1	D;V	Identify the study as developing and/or validating a multivariable prediction model, the target population, and the outcome to be predicted.	Title
Abstract	2	D;V	Provide a summary of objectives, study design, setting, participants, sample size, predictors, outcome, statistical analysis, results, and conclusions.	Abstract
**Introduction**				
Background and objectives	3a	D;V	Explain the medical context (including whether diagnostic or prognostic) and rationale for developing or validating the multivariable prediction model, including references to existing models.	Introduction, paragraphs 1-4
	3b	D;V	Specify the objectives, including whether the study describes the development or validation of the model or both.	Introduction, paragraph 4<br><br>"We therefore aimed to investigate if urinary LAM detection, along with other clinical variables readily available in high-burden settings, could be used to predict which HIV-positive patients admitted to hospital and diagnosed with TB patients were at high risk of early mortality in, and to externally validate the predictive tool."
**Methods**				
Source of data	4a	D;V	Describe the study design or source of data (e.g., randomized trial, cohort, or registry data), separately for the development and validation data sets, if applicable.	Methods, paragraph 1 (development), paragraph 10 (validation)
	4b	D;V	Specify the key study dates, including start of accrual; end of accrual; and, if applicable, end of follow-up.	Methods, paragraph 1 (October 2015 and September 2017) and Methods, paragraph 11 (LAM-RCT recruitment occurred between January 2013 and October 2014, and the MSF cohort between October 2013 and August 2015.)
Participants	5a	D;V	Specify key elements of the study setting (e.g., primary care, secondary care, general population) including number and location of centres.	Methods, paragraph 1 (hospital cohort)
	5b	D;V	Describe eligibility criteria for participants.	Methods, paragraph 2
	5c	D;V	Give details of treatments received, if relevant.	Methods, paragraph 3
Outcome	6a	D;V	Clearly define the outcome that is predicted by the prediction model, including how and when assessed.	Methods, paragraph 3 "The outcome was early mortality risk at 2-months after admission"
	6b	D;V	Report any actions to blind assessment of the outcome to be predicted.	NA
Predictors	7a	D;V	Clearly define all predictors used in developing or validating the multivariable prediction model, including how and when they were measured.	Methods, paragraph 4-5
	7b	D;V	Report any actions to blind assessment of predictors for the outcome and other predictors.	Methods, paragraph 2-3 Predictors all assessed prior to outcome occurring
Sample size	8	D;V	Explain how the study size was arrived at.	Methods, paragraph 3- Convenience sample size
Missing data	9	D;V	Describe how missing data were handled (e.g., complete-case analysis, single imputation, multiple imputation) with details of any imputation method.	Methods, paragraph 6 & 12
Statistical analysis methods	10a	D	Describe how predictors were handled in the analyses.	Methods, paragraph 6-7
	10b	D	Specify type of model, all model-building procedures (including any predictor selection), and method for internal validation.	Methods, paragraph 6-7
	10c	V	For validation, describe how the predictions were calculated.	Methods, paragraph 12

	10d	D;V	Specify all measures used to assess model performance and, if relevant, to compare multiple models.	Methods, paragraph 8-9, 13
	10e	V	Describe any model updating (e.g., recalibration) arising from the validation, if done.	None done
Risk groups	11	D;V	Provide details on how risk groups were created, if done.	Methods, paragraph 7 "High-, medium- and low-risk groups for mortality were then arbitrarily defined after plotting risk score against observed mortality, so the high-risk group accounted for most (>50%) deaths, and low-risk group accounted for as few deaths as possible"
Development vs. validation	12	V	For validation, identify any differences from the development data in setting, eligibility criteria, outcome, and predictors.	Methods, paragraph 11
**Results**				
Participants	13a	D;V	Describe the flow of participants through the study, including the number of participants with and without the outcome and, if applicable, a summary of the follow-up time. A diagram may be helpful.	Results, paragraph 1; Figure 1
	13b	D;V	Describe the characteristics of the participants (basic demographics, clinical features, available predictors), including the number of participants with missing data for predictors and outcome.	Results, paragraph 1; Table 1
	13c	V	For validation, show a comparison with the development data of the distribution of important variables (demographics, predictors and outcome).	Table 1, Figure 4
Model development	14a	D	Specify the number of participants and outcome events in each analysis.	Results, paragraph 1;, Figure 1
	14b	D	If done, report the unadjusted association between each candidate predictor and outcome.	Table 2
Model specification	15a	D	Present the full prediction model to allow predictions for individuals (i.e., all regression coefficients, and model intercept or baseline survival at a given time point).	Table 2
	15b	D	Explain how to the use the prediction model.	Results, paragraph 4-6;
Model performance	16	D;V	Report performance measures (with CIs) for the prediction model.	Figures 3 & 4; Results, paragraph 4, 6, 8-9
Model-updating	17	V	If done, report the results from any model updating (i.e., model specification, model performance).	NA
**Discussion**				
Limitations	18	D;V	Discuss any limitations of the study (such as nonrepresentative sample, few events per predictor, missing data).	Discussion, paragraphs 9-10
Interpretation	19a	V	For validation, discuss the results with reference to performance in the development data, and any other validation data.	Discussion, paragraph 11
	19b	D;V	Give an overall interpretation of the results, considering objectives, limitations, results from similar studies, and other relevant evidence.	Discussion, paragraphs 1-6
Implications	20	D;V	Discuss the potential clinical use of the model and implications for future research.	Discussion, paragraphs 5-6
**Other information**				
Supplementary information	21	D;V	Provide information about the availability of supplementary resources, such as study protocol, Web calculator, and data sets.	Data statement, supplement
Funding	22	D;V	Give the source of funding and the role of the funders for the present study.	Financial Disclosure Statement

*Items relevant only to the development of a prediction model are denoted by D, items relating solely to a validation of a prediction model are denoted by V, and items relating to both are denoted D;V.

We recommend using the TRIPOD Checklist in conjunction with the TRIPOD Explanation and Elaboration document.
