# Supplementary material for: Risk score for predicting mortality including urine lipoarabinomannan detection in hospital inpatients with HIV-associated tuberculosis in sub-Saharan Africa: Derivation and external validation cohort study
Source: PLoS Med. 2019 Apr 5;16(4):e1002776. doi: 10.1371/journal.pmed.1002776 (PMC6450614; doi:10.1371/journal.pmed.1002776)
Supplement: S3 Appendix — (PDF) [file pmed.1002776.s003.pdf]

S3 Appendix. List of ethics committees.

STAMP trial:
London School of Hygiene & Tropical Medicine Research Ethics Committee (UK)
Biomedical Research Ethics Committee, University of KwaZulu-Natal (South Africa)
College of Medicine Research Ethics Committee, University of Malawi (Malawi)

LAM-RCT:
Human Research Ethics Committee of The Faculty of Health Sciences, University of Cape Town (South Africa)
Biomedical Research Ethics Committee, University of KwaZulu-Natal (South Africa)
University of Zambia (UNZA) Biomedical Research Ethics Committee (Zambia)
Joint Parirenyatwa Hospital and College of Health Sciences Research Ethics Committee, University of Zimbabwe (Zimbabwe)
National Health Research Ethics Review Committee (Tanzania)

MSF Homa Bay cohort:
KEMRI/Scientific and Ethics Review Committee (Kenya)
Comité de Protection des Personnes (CPP), Saint Germain en Laye, (France)
