# Supplementary figures and images for: Risk score for predicting mortality including urine lipoarabinomannan detection in hospital inpatients with HIV-associated tuberculosis in sub-Saharan Africa: Derivation and external validation cohort study
Source: PLoS Med. 2019 Apr 5;16(4):e1002776. doi: 10.1371/journal.pmed.1002776 (PMC6450614; doi:10.1371/journal.pmed.1002776)

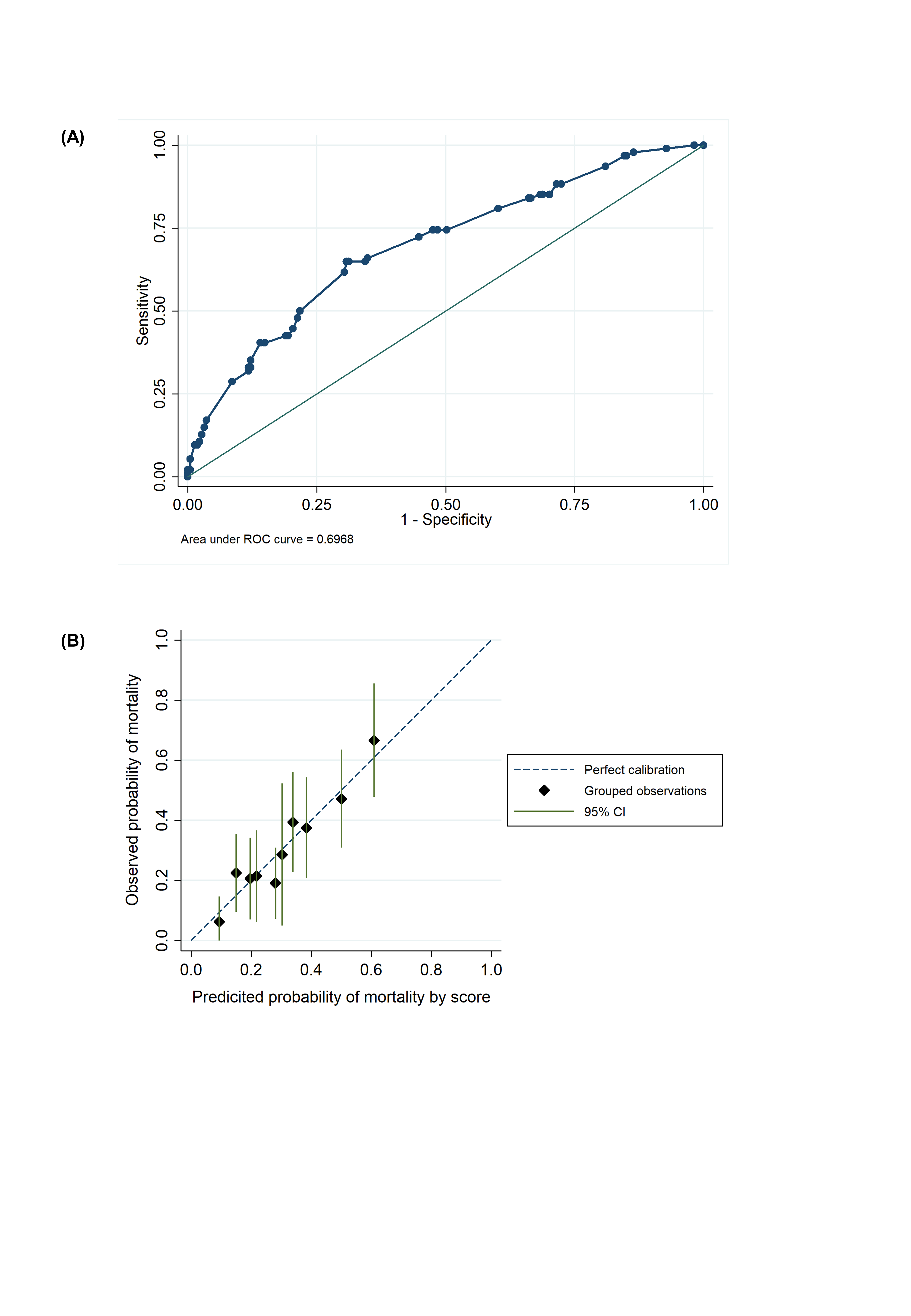

Supplement: S1 Fig — (A) Receiver operator curve of the predictive model: area under the curve = 0.70 (95% CI 0.63–0.76). (B) Calibration plot of observed probability of mortality plotted against predicted probability of mortality by the risk score multivariable regression model, with variables grouped into deciles based on predicted probability, and 95% CIs. Black dashed line shows perfect prediction. Hosmer-Lemeshow statistic p = 0.78. (TIF) [file pmed.1002776.s004.tif]

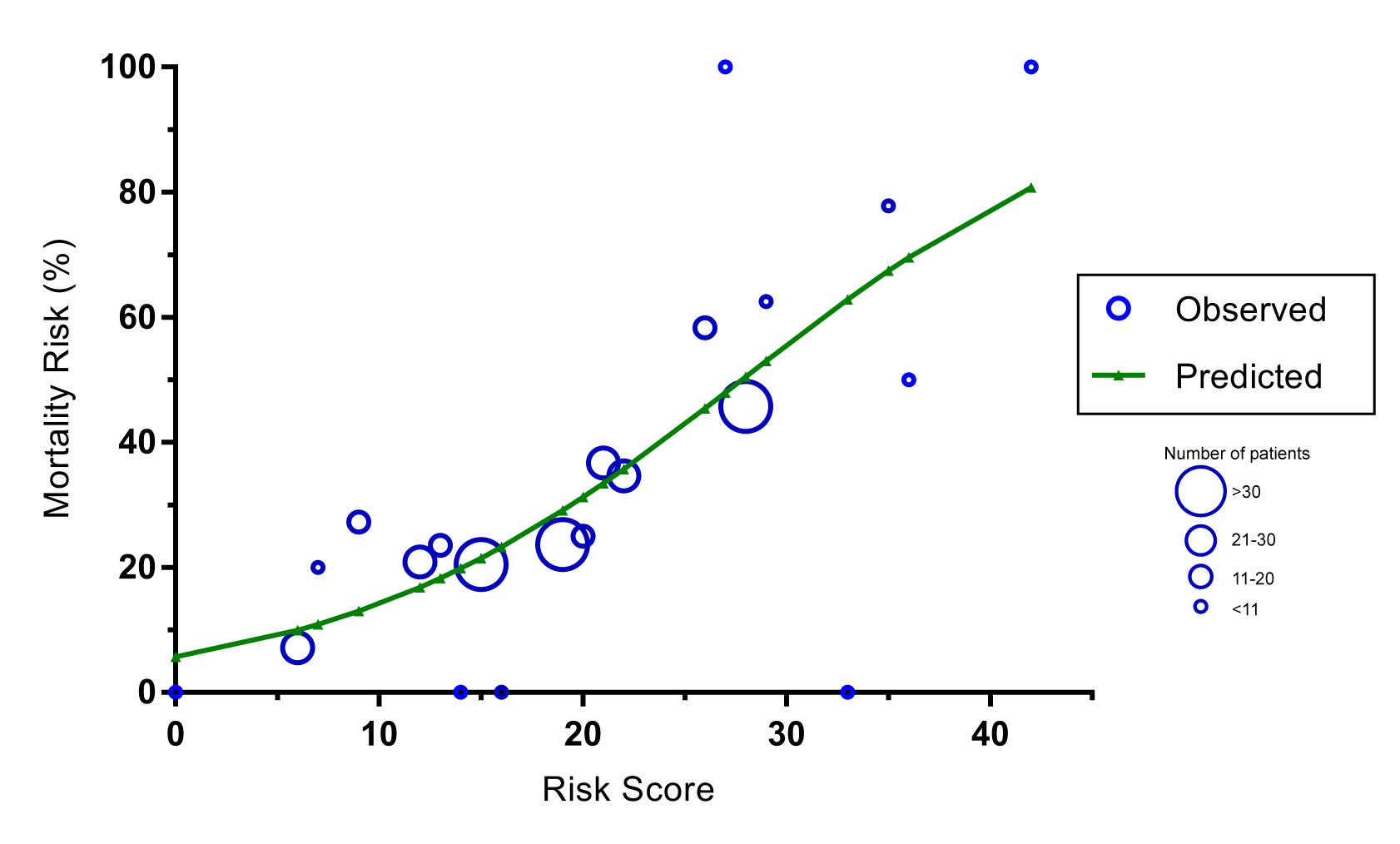

Supplement: S2 Fig — The size of the blue circles representing observed mortality risk is proportional to the number of patients with that score. Predicted mortality risk is represented by the green line/triangles. (TIF) [file pmed.1002776.s005.tif]

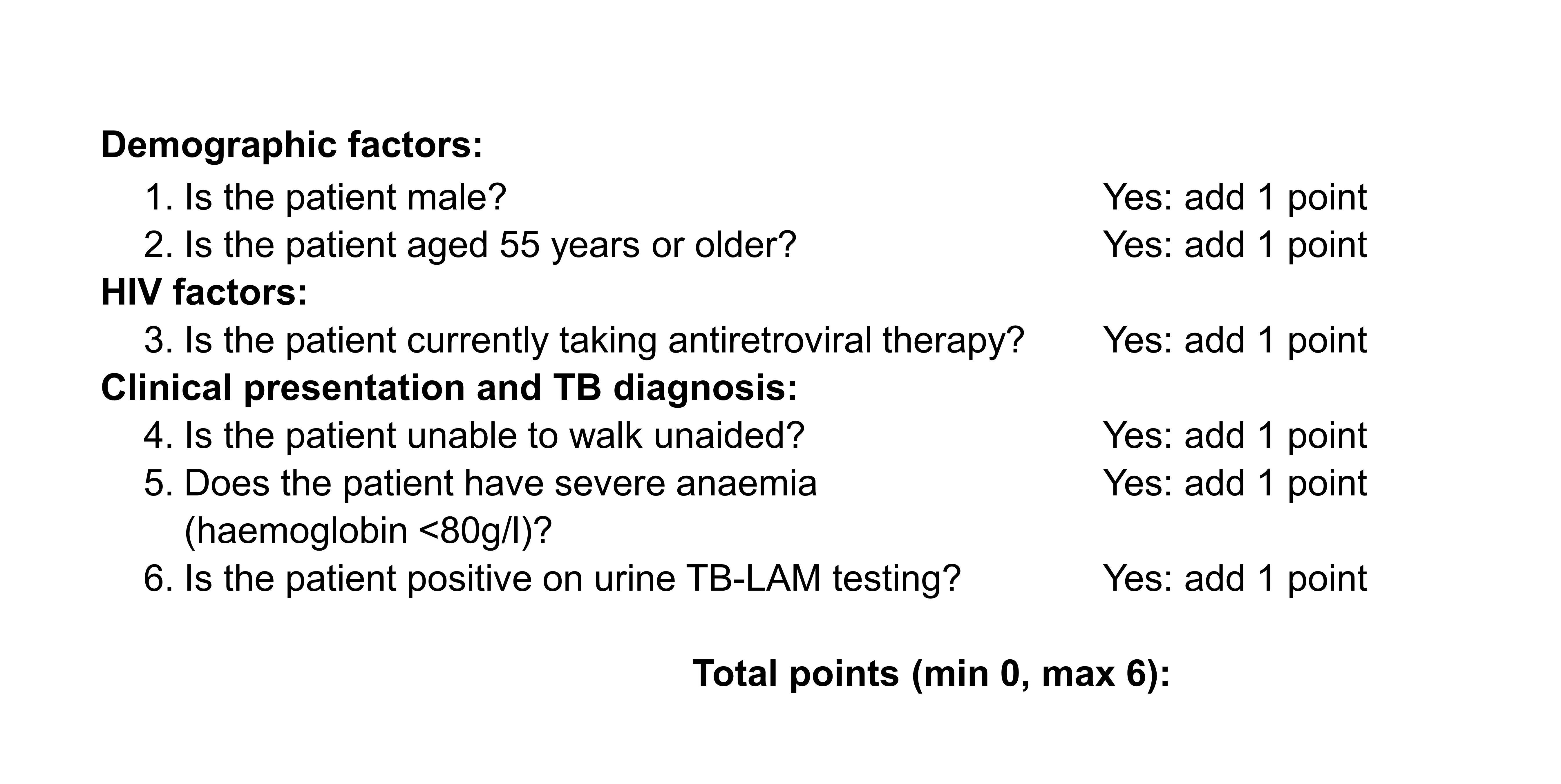

Supplement: S3 Fig — (TIF) [file pmed.1002776.s006.tif]

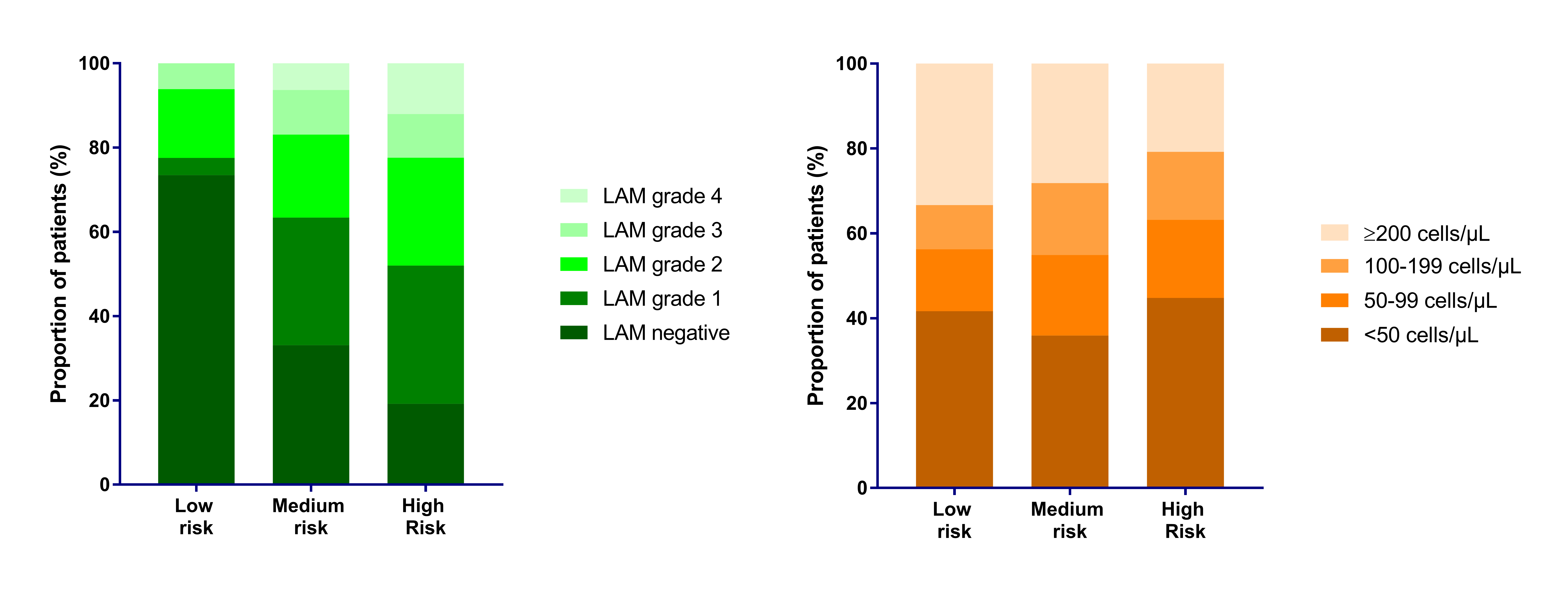

Supplement: S4 Fig — (TIF) [file pmed.1002776.s007.tif]

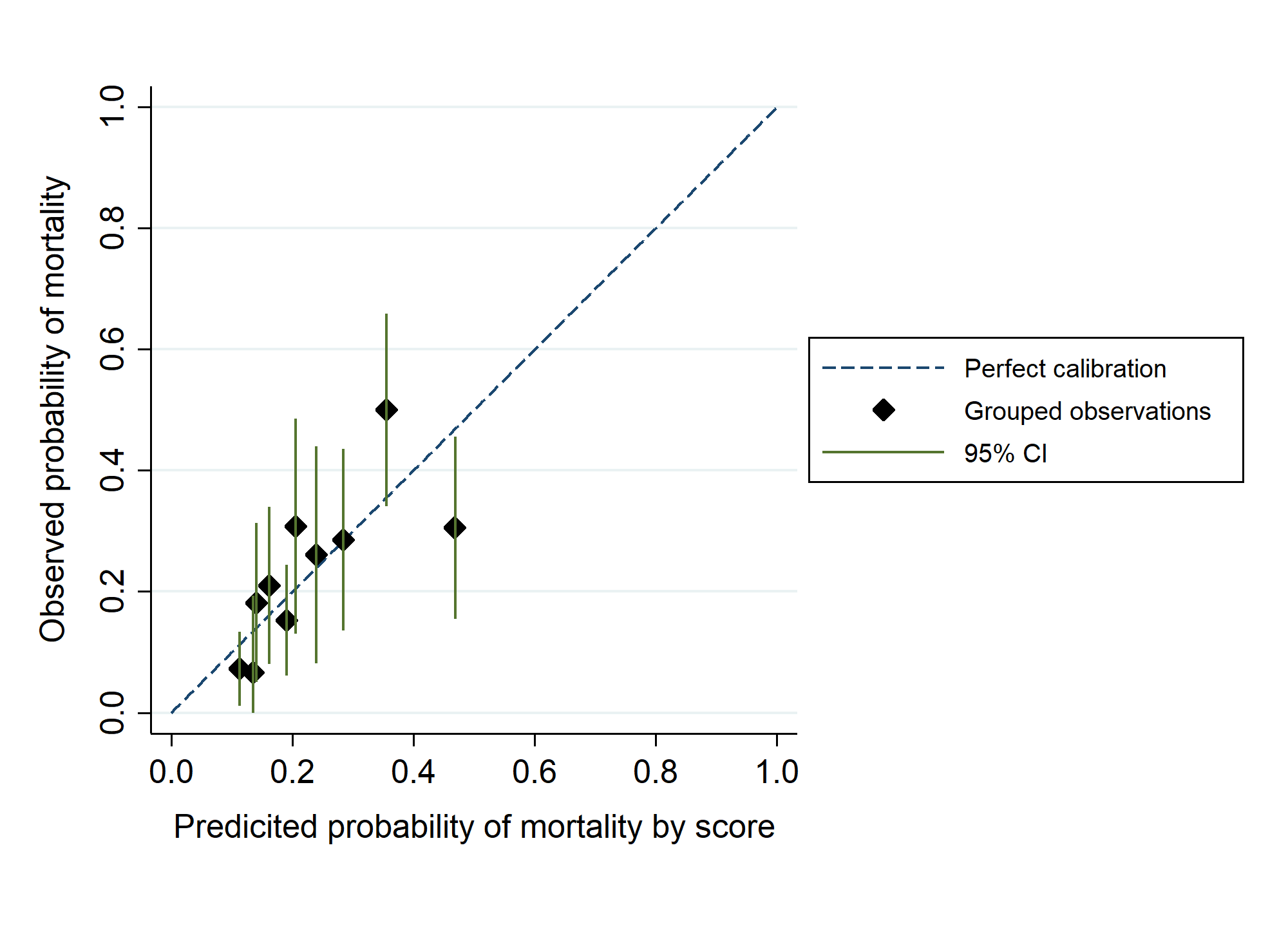

Supplement: S5 Fig — Plot shows the observed compared to expected probability of risk for the external validation cohort as deciles based on risk score, with 95% CIs. Hosmer-Lemeshow statistic p = 0.13. c-Statistic (or area under the receiver operator curve) was 0.68 (95% CI 0.61–0.74). Dotted line represents perfect prediction. (TIF) [file pmed.1002776.s008.tif]
