# Supplementary material for: Risk score for predicting mortality including urine lipoarabinomannan detection in hospital inpatients with HIV-associated tuberculosis in sub-Saharan Africa: Derivation and external validation cohort study
Source: PLoS Med. 2019 Apr 5;16(4):e1002776. doi: 10.1371/journal.pmed.1002776 (PMC6450614; doi:10.1371/journal.pmed.1002776)
Supplement: S1 Table — (PDF) [file pmed.1002776.s009.pdf]

S1 Table. Univariable analysis of continous variables and assocations with mortality.

	Fitted f rational polynomial function	Odds ratio (OR), 95% confidence interval	p-value
**Age (years)[a]**			
Linear		1.02 (1.00 – 1.05)	0.0485
Age^3<br>Age^3	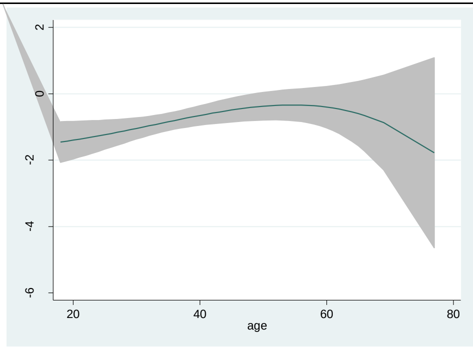	1.00 (1.00 – 1.00)<br>1.00 (1.00 – 1.00)	0.0526
**Weight (kgs)b**			
Linear		0.98 (0.96 – 1.00)	0.0486
Weight^-2<br>Weight^-2	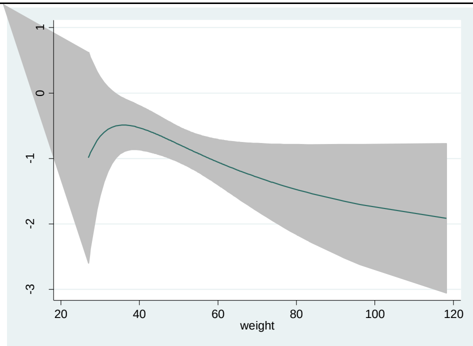	0<br>0	0.0897
**CD4 count[c]**			
Linear		1.00 (1.00 – 1.00)	0.0158
CD4 count^-2<br>CD4 count	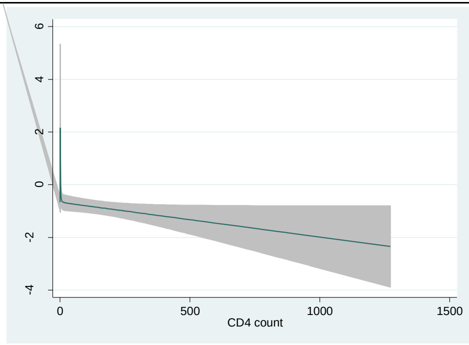	16.7 (0.65 – 430.71)<br>1.00 (1.00 – 1.00)	0.0055
**Haemoglobin (g/L)[d]**			
Linear		0.98 (0.97 – 0.99)	0.0002
Haemoglobin^3<br>Haemoglobin^3	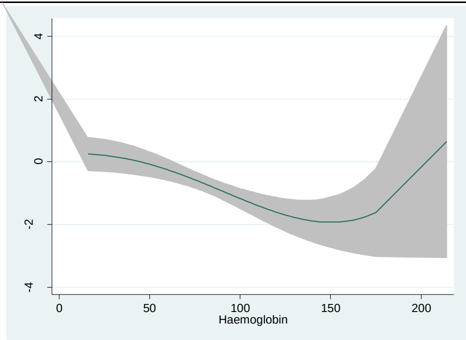	1.00 (1.00 – 1.00)<br>1.00 (1.00 – 1.00)	0.0001

[a] This function has a small increase to 55 years, then a steeper increase

[b] This gives a “U-shaped” function and has an increase until 35kg, then a sharper decrease until 60kg, and then a less steep increase

[c] This function has a sharp peak in the low values (<20 cell/microL), followed by a linear decrease

[d] This is a “U-shaped” function and has a decrease until about 150 g/L, followed by an increase.
