# Supplementary material for: Risk score for predicting mortality including urine lipoarabinomannan detection in hospital inpatients with HIV-associated tuberculosis in sub-Saharan Africa: Derivation and external validation cohort study
Source: PLoS Med. 2019 Apr 5;16(4):e1002776. doi: 10.1371/journal.pmed.1002776 (PMC6450614; doi:10.1371/journal.pmed.1002776)
Supplement: S2 Table — Number of patients surviving and patients dying, and observed and predicted mortality risk for (A) the full clinical risk score (based on the regression coefficients) and (B) the simplified risk score. (PDF) [file pmed.1002776.s010.pdf]

S2 Table. Risk score and mortality data

Number of survivors, patients dying, observed and predicted mortality risk for **A)** 'full' mortality predictor score (based on the regression coefficients) and **B)** simplified mortality risk score.

A)

Risk score category	Proportion of patients	Survived	Died	Observed mortality	Predicted Mortality
**0-5**	1.3	4	0	0.0	7.7
**6-10**	14.0	38	6	13.6	12.1
**11-15**	27.3	68	18	20.9	18.4
**16-20**	17.8	44	12	21.4	27.0
**21-25**	17.8	36	20	35.7	37.8
**26-30**	17.8	27	29	51.8	49.9
**31-35**	3.2	3	7	70.0	62.0
**>35**	1.0	1	2	66.7	72.8
**Low**	15.2	42	6	12.5	9.7
**Medium**	45.1	112	30	21.1	23.0
**High**	39.7	67	58	46.4	45.3

Mortality risk groups were defined as low risk (10 points or fewer), medium risk (11 to 20 points) or high risk (more than 20 points)

B)

Risk score or risk score category	% of patients	Survived	Died	Observed mortality	Predicted Mortality
**0**	1.3	4	0	0.0	5.6
**1**	14.0	38	6	13.6	10.7
**2**	29.2	74	18	19.6	19.5
**3**	33.7	74	32	30.2	32.7
**4**	17.8	27	29	51.8	49.5
**5**	3.8	4	8	66.7	66.3
**6**	0.3	0	1	100.0	79.9
**Low**	15.2	42	6	12.5	10.5
**Medium**	29.2	74	18	19.6	21.7
**High**	55.6	105	70	40.0	39.5

Mortality risk groups were defined as low risk (1 point or fewer), medium risk (2 points) or high risk (more than 2 points)
