# Supplementary material for: Risk score for predicting mortality including urine lipoarabinomannan detection in hospital inpatients with HIV-associated tuberculosis in sub-Saharan Africa: Derivation and external validation cohort study
Source: PLoS Med. 2019 Apr 5;16(4):e1002776. doi: 10.1371/journal.pmed.1002776 (PMC6450614; doi:10.1371/journal.pmed.1002776)
Supplement: S3 Table — (PDF) [file pmed.1002776.s011.pdf]

S3 Table. C-statistic, 95% confidence intervals and Hosmer-Lemshow test for final model and risk scores in derivation and validation cohorts.

Cohort/model	C-statistic	95% Conf. Interval	Hosmer-Lemshow test p-value
Derivation cohort full model	0.7299	0.66957 - 0.79015	0.4527
Derivation cohort full score	0.6968	0.63280 - 0.76077	0.7813
Derivation cohort simple score	0.6471	0.59337 - 0.70092	0.4956
Validation cohort full model	0.6659	0.60062 - 0.73123	0.2334
Validation cohort full score	0.6770	0.61307 - 0.74083	0.1315
Validation cohort simple score	0.6605	0.59756 - 0.72336	0.2269
